# Ethanolic extract of *Commiphora myrrha* gum-resin promotes therapeutic compound accumulation in *Achillea fragrantissima* under *in vitro* culture

**DOI:** 10.3389/fpls.2025.1714322

**Published:** 2026-01-28

**Authors:** Mohamed S. Khattab, Munirah F. Al Dayel, Fadia El Sherif

**Affiliations:** 1 Department of Clinical Pharmacy, Suez Canal University, Ismailia, Egypt; 2 Department of Biological Sciences, College of Science, King Faisal University, Al Ahsa, Saudi Arabia

**Keywords:** fragrant yarrow, myrrh, biostimulant, effect compound, tissue culture, explant type

## Abstract

**Introduction:**

*Achillea fragrantissima* is valued for its broad spectrum of secondary metabo-lites with notable therapeutic potential. This study examined the influence of an ethanolic extract of Commiphora myrrha gum-resin on in vitro shoot growth, callus formation, and the effect of compound composition in A. fragrantissima.

**Material and methods:**

Shoot-tip and root-segment explants were cultured on Murashi-ge and Skoog (MS) medium supplemented with filter-sterilized *C. myrrha* ethanolic extract at 0.5, 1.0, and 2.0 g·L^-1^, alongside control treatment. Plant responses, including growth parameters, callus induction percentage and biomass, photosynthetic pigments, and me-tabolite composition, were assessed after one month of in vitro culture. GC–MS analysis was performed on the *C. myrrha* extract and on methanolic extracts of *A. fragrantissima* plantlets and callus tissues obtained from different treatments.

**Results:**

GC–MS analysis of the ethanolic extract of *C. myrrha* identified 66 phytochemi-cal compounds dominated by sesquiterpenes—particularly 2,5,8-trimethyl-1-nonen-3-yn-5-ol, curzerene, myrcenol, germa-cra-1(10),4,11(13)-trien-12-oic acid, and several oxygenated sesquiterpenes—indicating a rich pool of bioactive, growth-promoting constituents. Shoot-tip and root-segment ex-plants showed the strongest responses to *C. myrrha* extract at specific concentrations. The highest concentration tested (2.0 g·L^-1^) produced the most shoots and the greatest accu-mulation of photosynthetic pigments, including total chlorophyll and carotenoids. In contrast, the lowest concentration (0.5 g·L^-1^) promoted the longest shoots, the highest fresh weight, and the greatest number of leaves. Callus formation from shoot tips was also highest at 0.5 g•L^-1^, while root-derived callus reached full induction at both 0.5 and 2.0 g·L^-1^. Both shoot-tip plantlets and root-derived callus showed notable modifications in their bioactive constituents. Several bioactive constituents were notably elevated (e.g., de-sulphosinigrin, thymidine, 4H-pyran-4-one derivatives, and fatty acid derivatives).

**Discussion:**

Distinct tissue-specific patterns emerged, shoot tips accumulated a wider range of aromatic and therapeutic compounds, whereas callus tissue was enriched in fat-ty acids. These findings suggest that *C. myrrha* extract functions as a natural biostimulant and elicitor, providing a sustainable approach for producing metabolite-rich *A. fragrantis-sima* material with potential pharmacological applications.

## 1 Introduction

*Achillea fragrantissima* L. (Asteraceae), commonly known as fragrant yarrow, is a perennial herb native to the Middle East, North Africa, and the Mediterranean basin (Goda et al., 2023). The plant has long been valued in traditional medicine, largely due to its rich secondary metabolites, including essential oils, flavonoids, and terpenes. These compounds contribute to its documented antioxidant, antimicrobial, anti-inflammatory, and anticancer properties (Alsayed et al., 2024; Tawfik et al., 2024). *In vitro* culture techniques provide a practical solution for producing uniform, contaminant-free plant material regardless of season or geography (Ozyigit et al., 2023; Usman et al., 2025). Such approaches allow researchers to control growth conditions and apply treatments that can stimulate metabolic pathways. Among these treatments, using natural plant extracts as elicitors has become a highly effective way to boost the production of secondary metabolites. Plant extract–based elicitors can mimic natural biotic or abiotic stress signals, triggering defense-related enzymes and hormone-like pathways. This activation enhances the plant’s production of valuable bioactive compounds (Sood and Sood, 2020; Abdulhafiz et al., 2022; Fazili et al., 2022; Kanso et al., 2022; Mohaddab et al., 2022; Jampílek and Kráľová, 2023). Furthermore, the use of natural plant extracts can improve the plant’s resilience to environmental stressors, thereby increasing both the quantity and quality of the bioactive compounds. This makes them ideal candidates for exploring sustainable methods of enhancing bioactive compounds, with potential applications in agriculture, herbal medicine, and the development of functional foods and nutraceuticals (El Sherif et al., 2020; Yap et al., 2021). *Commiphora myrrha* (myrrh), the gum-resin traditionally used in ethnomedicine across the Arabian Peninsula, contains a chemically diverse mixture of terpenoids including mono-, sesqui-, and triterpenoids along with oxygenated derivatives and volatile oils (Batiha et al., 2023). Many of these constituents have been implicated in defense signaling and stress response pathways in plants, suggesting that myrrh resin could act as a natural elicitor in tissue culture systems (Hilgers et al., 2021). Ethanolic extracts of medicinal plants, rich in phenolics, flavonoids, and alkaloids, have been used as natural biostimulants under *in vitro* conditions to enhance cell division, shoot proliferation, and plant growth (El Sherif et al., 2020). In particular, ethanolic extracts of *Moringa oleifera* leaves (MLE) have been shown to improve morphogenesis, plantlet vigor, and stress tolerance in plant cultures, both under *in vitro* and field conditions, likely due to their bioactive compounds that mimic or enhance endogenous plant hormones (Alkuwayti et al., 2020; El Sherif et al., 2020; Yap et al., 2021). These effects are attributed to growth-promoting compounds that mimic or enhance plant hormones, making ethanolic extracts a promising, eco-friendly biostimulant. Based on this, we postulated that when added to culture media for *A. fragrantissima*, ethanolic extracts of *C. myrrha* would serve as efficient elicitors and biostimulants. We sought to establish whether extract treatments could enhance callus induction and shoot growth *in vitro*, while optimizing the amount of photosynthetic pigment. The metabolite profiles of the shoot plantlets and callus produced from the roots were identified by Gas chromatography–mass spectrometry (GC-MS). The ultimate goal of this study is to develop an extract-based, sustainable strategy for increasing the quantity and quality of pharmacologically significant metabolites in *A. fragrantissima*.

## 2 Materials and methods

### 2.1 Plant material and resin source

*C. myrrha* gum-resins were obtained from Earth Circle Organics (USA). *A. fragrantissima* seeds were collected from Wadi Harqan, Al-Quraynah, Riyadh Region, Saudi Arabia (2024). This species is taxonomically identified by Prof. Dr Mona Alwheeby, Department of Botany and Microbiology, College of Science, King Saud University, Riyadh, Saudi Arabia. Voucher information and seed handling followed standard propagation protocols to ensure the authenticity of the species and the viability of the seeds.

### 2.2 Preparation of *C. myrrha* ethanolic extract

Ethanolic extracts of *C. myrrha* gum-resin were prepared according to the method described in Hassanzadeh-Taheri et al. (2021). After extraction, the ethanol was evaporated, and the dried extract was resuspended in 10% ethanol to obtain a 1.0 g/mL stock solution. The stock solution was sonicated to ensure proper dispersion of the extract. The sonicated stock was subsequently filter-sterilized with a 0.22 μm syringe filter under aseptic conditions before being incorporated into the autoclaved culture media to achieve the desired final concentrations (0.5 g·L^-1^, 1.0 g·L^-1^, and 2.0 g·L^-1^) of extract, according to the treatment. An appropriate volume of 10% ethanol was incorporated into the control (consisting of MS medium supplemented with 0.2 mg·L^-1^ benzylaminopurine,BAP for shoot tip culture, or 0.7 mg·L^-1^ BAP combined with 0.5 mg·L^-1^ indole-3-acetic acid, IAA for callus culture). The final concentrations of ethanol in the media of all treatment groups are 0.02%.

### 2.3 *In vitro* shoot multiplication of shoot-tip explants and elicitation

Shoot-tip explants (0.5–1.0 cm long) of *A. fragrantissima* were excised from one-month-old *In vitro* germinated seedlings. These explants were subcultured into 200 mL culture vessels containing 30 mL Murashige and Skoog (MS) basal medium (6 g·L^-1^ agar, 3% w/v sucrose) supplemented with 0.2 mg·L^-1^ BAP (Goda et al., 2023). Filter-sterilized *C. myrrha* gum-resin ethanolic extract was added to autoclaved medium cooled to 47°C to achieve final concentrations of 0.0 (control), 0.5, 1.0, and 2.0 g·L^-1^. Each treatment consisted of 20 replicates (ten culture vessels, each holding two shoot tips). Cultures were incubated at 24 ± 2°C under a 16-h photoperiod with a light intensity of ~4000 lux (El Sherif et al., 2025). After one month, the following growth parameters were recorded: fresh weight per explant (g), longest shoot length (cm), number of shoots and number of leaves per explant, as well as callus percentage (%). Plantlets from each treatment were air-dried and extracted with methanol for GC–MS analysis.

### 2.4 Callus induction of root explants and elicitation

Root segment explants (0.5–1.0 cm) of *A. fragrantissima* were excised from one-month-old *in-vitro* seedlings and cultured on MS medium supplemented with 0.7 mg·L^-1^ BAP and 0.5 mg·L^-1^ IAA (Goda et al., 2023). *C. myrrha* gum-resin ethanolic extract was filter-sterilized and added to cooled, autoclaved medium at final concentrations of 0.0 (control), 0.5, 1.0, and 2.0 g·L^-1^. Thirty milliliters of medium were poured into 92 × 16 mm Petri dishes, with four root explants per dish. Each treatment included 12 replicates (petri dishes). Cultures were incubated in the dark at 24 ± 2°C (El Sherif et al., 2025). After one month, two parameters were measured: the percentage of callus induction and the callus fresh weight per explant (g). Callus tissue was air-dried prior to extraction with methanol for GC–MS analysis.

### 2.5 Photosynthetic pigment analysis

Photosynthetic pigments—chlorophyll a, chlorophyll b, and carotenoids—were quantified from leaf samples (explant from shoot multiplication treatment after one month of culture) of three randomly selected explants per treatment. The quantification was performed using the colorimetric method described by A.O.A.C (1984). Pigment contents were expressed as mg·100 g^-1^ fresh weight.

### 2.6 GC-MS analysis

GC–MS profiling was performed at the Department of Chemistry, College of Science, King Faisal University. *C. myrrha* resin was extracted with 80% ethanol following Hassanzadeh-Taheri et al. (2021).

*A. fragrantissima* plantlets (derived from shoot tip explants) and callus tissues were extracted with 99% methanol following the method described by (El-Ashmawy et al., 2016) Plantlets from each treatment were air-dried and subjected to methanolic extraction for GC–MS analysis. Three samples were collected per treatment for the analysis. Analyses were performed using a Shimadzu GC-MS QP2010 Plus equipped with an AOC-20i auto-sampler. Separation was achieved using an RTX^®^-5Sil MS capillary column (5% diphenyl–95% dimethylpolysiloxane). Constituents were identified by comparing mass spectra and retention indices with reference data, while relative percentage composition was calculated from GC peak areas (Lee et al., 2018) (Lee et al., 2018) with slight modifications.

### 2.7 Statistical analysis and experimental design

The experiment was arranged in a Randomized Complete Block Design. Statistical analysis was performed using one-way ANOVA, followed by the Tukey–Kramer *post-hoc* test to compare treatment means. A significance level of p ≤ 0.05 was applied. All analyses were conducted using GraphPad Prism software (version 8; GraphPad Software, San Diego, CA, USA).

## 3 Results

### 3.1GC–MS profile of *C. myrrha* gum-resin ethanolic extract

GC–MS analysis of the ethanolic extract of *C. myrrha* resin revealed 66 compounds eluting between 9.44 and 22.40 minutes (**Table 1**). The dominant peak, representing 15.71% of the total area, corresponded to 2,5,8-trimethyl-1-nonen-3-yn-5-ol, an oxygenated non-terpene. Sesquiterpenes were well represented, including curzerene (8.24%), germacrene B (2.81%), valencene (1.69%), α-copaene (0.83%), and caryophyllene (0.44%). Oxygenated sesquiterpenes such as (−)-(1R,2S)-2,3-epoxy-2-(methoxymethyl)-6,6-dimethylbicyclo[3.1.1]heptane (6.86%) and germacra-1(10),4,11(13)-trien-12-oic acid (4.04%) were also notable. Monoterpene hydrocarbons (e.g., r(+)-limonene, β-pinene) were present at very low levels (~0.02% each), whereas the oxygenated monoterpene myrcenol contributed 6.71% of the total composition. Altogether, the extract displayed a complex mix of mono- and sesquiterpenes, oxygenated derivatives, and minor aromatics—compounds often implicated in plant defense and signaling.

**Table 1:** Phytochemical composition of ethanol extracts from *C. myrrha* gum -resin by GC-MS.

Peak	Phytochemical compounds	RT, min	Area, %	Molecular formula	Molecular weight (g/mol)
1	3,5-xylenol	9.439	0.04	C10H16	136.23
2	R(+)-Limonene	10.149	0.02	C10H16	136.23
3	(-)-Elema-1,3,11(13)-trien-12-ol	10.392	0.04	C15H24O	220.35
4	p-Cumic aldehyde	10.704	0.01	C10H12O	148.2
5	2-Methyl-6-methylene-2,7-octadienal	11.756	0.01	C10H14O	150.22
6	Cis,cis,trans-3,3,6,6,9,9-hexamethyl-tetracyclo[6.1.0.0(2,4).0(5,7)]nonane	12.077	0.01	C15H24	204.35
7	alpha.-Terpinene	12.375	0.18	C10H16	136.23
9	Naphthalene, 1,2,3,4,4a,5,6,8a-octahydro-7-methyl-4-methylene-1-(1-methylethyl)-, (1.alpha.,4a.alpha.,8a.alpha.)	12.648	0.03	C15H24	204.35
11	Furan, 5-methyl-2,2’-methylenedi-	12.845	0.01	C10H10O2	162.19
12	(-)-alpha-Cubebene	13.015	0.01	C15H24	204.35
13	Copaene	13.205	0.07	C15H24	204.35
14	Curzeren	13.307	0.36	C15H22	202.34
15	Germacrene B	13.433	2.81	C15H24	204.35
16	β-Patchoulene	13.525	0.02	C15H24	204.35
17	Caryophyllene	13.735	0.44	C15H24	204.35
18	β-Pinene	13.976	0.02	C10H16	136.23
20	Cadina-1(10),4-diene	14.182	0.03	C15H24	204.35
22	Humulene	14.428	0.13	C15H24	204.35
23	1H-Cycloprop[e]azulene, decahydro-1,1,4,7-tetramethyl-	14.516	0.07	C15H26	206.37
24	Azulene, 1,2,3,4,5,6,7,8-octahydro-1,4-dimethyl-7-(1-methylethenyl)-, (1S,4S,7R)	14.584	0.17	C15H24	204.35
26	α-Copaene	14.783	0.83	C15H24	204.35
27	(-)-(1R,2S)-2,3-epoxy-2-(methoxymethyl)-6,6-dimethylbicyclo[3.1.1]heptane	14.992	6.86	C12H20O2	196.29
28	Menthofuran	15.061	1.51	C10H14O	150.22
29	Cyclohexanemethanol, 4-ethenyl-.alpha.,.alpha.,4-trimethyl-3-(1-methylethenyl)-, [1R-(1.alpha.,3.alpha.,4.beta.)]-	15.255	0.1	C15H26O	222.37
31	.gamma.-Elemene	15.796	1.57	C15H24	204.35
32	Benzofuran, 4,5,6,7-tetrahydro-3,6-dimethyl	15.952	0.48	C10H14O	150.22
33	Caryophyllene oxide	16.169	0.12	C15H24O	220.35
34	(-)-(R)-ipsdienol	16.265	0.1	C15H26O	222.37
35	1-Naphthalenol, 1,2,3,4,4a,7,8,8a-octahydro-1,6-dimethyl-4-(1-methylethyl)-, [1R-(1.alpha.,4.beta.,4a.beta.,8a.beta.)]-	16.474	0.17	C15H26O	222.37
36	2,5,8-trimethyl-1-nonen-3-YN-5-ol	16.686	15.71	C12H20O	180.29
37	Curzerene	16.823	8.24	C15H22	202.34
38	Naphthalene, 1,2,3,4,4a,5,6,8a-octahydro-7-methyl-4-methylene-1-(1-methylethyl)-, (1.alpha.,4a.beta.,8a.alpha.)-	16.928	3.67	C15H24	204.35
39	1,4-Methano-1H-indene, octahydro-4-methyl-8-methylene-7-(1-methylethyl)-	17.01	0.3	C12H18	162.27
40	Valencene	17.306	1.69	C15H24	204.35
41	Bicyclo[3.1.1]hept-2-ene-2-carboxaldehyde,6,6-dimethyl-,(1S)-	17.306	1.38	C10H14O	150.22
42	Azulene, 1,2,3,4,5,6,7,8-octahydro-1,4-dimethyl-7-(1-methylethenyl)-, (1S,4S,7R)-	17.492	2.95	C15H26	206.37
43	Myrcenol	17.643	6.71	C10H18O	154.25
44	1H-Cycloprop[e]azulen-7-ol, decahydro-1,1,7-trimethyl-4-methylene-, [1ar-(1aalpha,4aalpha,7beta,7abeta,7balpha)]-	17.847	1.08	C15H24O	220.35
45	Guaiol	18.018	0.47	C15H26O	222.37
46	Oxalic acid, hexyl 2-methylphenyl ester	18.559	1.89	C15H20O4	264.32
47	alpha-Curcumene	18.683	1.84	C15H22	202.34
49	4-[2′-methyl-5′-(2″-methyl-2″-propen-1″-YL)-2′-cyclopenten-1′-yliden]butan-2-one	19.141	0.4	C15H24	204.35
50	Guaia-1(10),11-diene	19.216	2.65	C15H24	204.35
51	Germacra-1(10),4,11(13)-trien-12-oic acid	19.653	4.04	C15H22O2	234.34
52	Guaia-1(10),11-diene	19.714	3.38	C15H24	204.35
53	Cis,cis,trans-3,3,6,6,9,9-hexamethyl-tetracyclo[6.1.0.0(2,4).0(5,7)]nonane	19.989	0.65	C15H24	204.35
54	3-methyl-1-(4-methylphenyl)-1-phenyl-1,2-butanediol	20.07	3.53	C18H22O2	270.37
58	6-Isopropenyl-4,8a-dimethyldecahydro-1-naphthalenol	20.916	2.05	C15H26O	222.37
60	4,8,13-Duvatriene-1,3-diol	21.298	1.71	C15H24O2	236.35
64	Naphthalene, 1,2,3,4-tetrahydro-6-methoxy	22.092	0.66	C11H14O	162.23
66	2-Isopropenyl-4,4,6b-trimethyl-4-.	22.411	0.49	C15H24	204.35

### 3.2 Effect of *C. myrrha* gum -resin ethanolic extract (g·L^-1^) concentrations on shoot induction and growth

**Figure 1:**
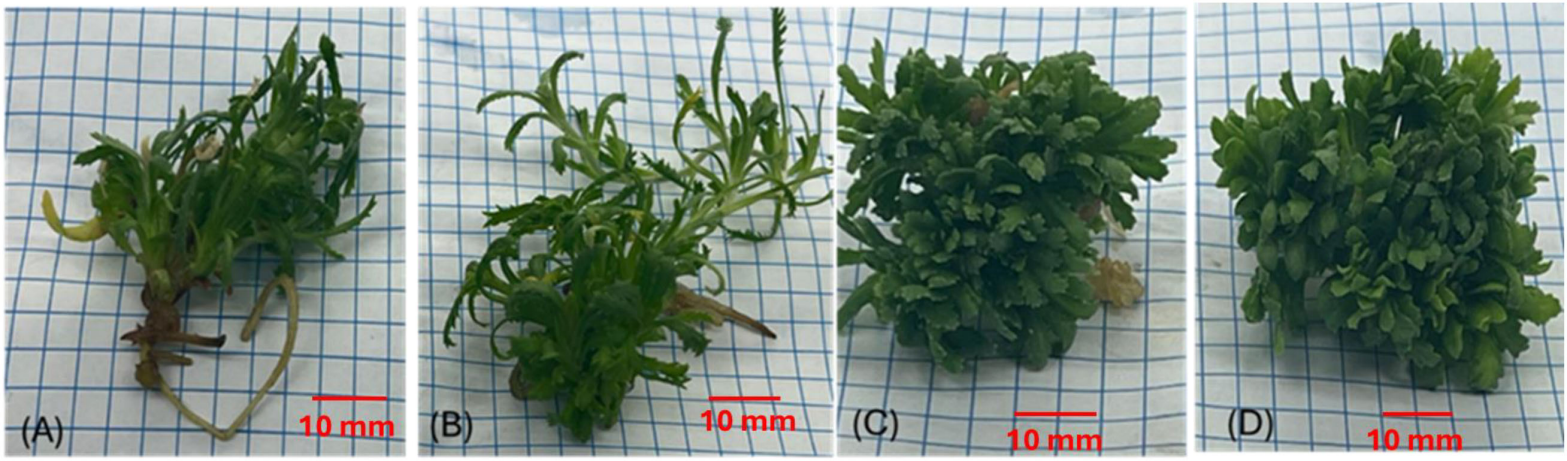
Effect of *C*. *myrrha* gum-resins ethanolic extract (g·L^-1^) concentrations on multiple shoots induction of *A*. *fragrantissima*. **(A)** control **(B)** 0.5 g·L^-1^, **(C)** 1.0 g·L^-1^, and **(D)** 2.0 g·L^-1^ are concentrations of *C*. *myrrha* gum-resin ethanolic extract respectively.

The effect of *C. myrrha* gum-resin ethanolic extract on multiple shoot induction in *A. fragrantissima* was clearly concentration-dependent (**Table 2**, **Figure 1**). In the control treatment (**Figure 1A**), shoot induction was minimal, with the shortest shoots (1.75 cm), lowest fresh weight (0.717 g), fewest leaves per explant (39.17), and the lowest shoot number (11.83 per explant), while callus formation was also minimal (20.83%). At 0.5 g·L^-1^, explants exhibited improved growth, with longer shoots (2.83 cm), higher fresh weight (0.986 g), more leaves per explant (72.86), and increased shoot number (17.17 per explant), while callus formation peaked at 45.83% (**Figure 1B**). Increasing the concentration to 1.0 g·L^-1^ significantly enhanced shoot proliferation (20.71 shoots per explant), fresh weight (1.30 g), and leaf number (88.75), although callus formation slightly declined to 35.71% compared with the control (**Figure 1C**). The highest concentration tested, 2.0 g·L^-1^, produced the greatest shoot number (27.5 per explant) and leaf number (100.5), significantly higher than the control, while shoot length (2.75 cm) and fresh weight (1.23 g) were slightly lower than at 1.0 g·L^-1^. Similarly, the callus formation also decreased to 25%, which is lower than that observed for 1.0 g·L^-1^ treatment (**Figure 1D**).

**Table 2:** Effects of *C. myrrha* gum-resin ethanolic extract (g·L^-1^) on shoot induction of *A. fragrantissima*. Shoot parameter values represent the mean of 20 replicates (10 culture vessels containing two shoots each).

*C. myrrh* (g·L^-1^)	Length of the longest shoot [cm]	Explant’s fresh weight [g]	No. of leaves/explant [n]	No. of shoot/explant [n]	Callus %
Control	1.75 ± 0.28 b	0.717± 0.0.32 c	39.17± 0.33 d	11.83± 0.50 c	20.83 ± 0.57 d
0.5	2.83± 0.14 a	0.986 ± 0.15 b	72.86± 0.45 c	17.17 ± 0.46 bc	45.83± 0.22 a
1	2.357 ± 0.06 ab	1.3 ± 0.28 a	88.75 ± 0.29 b	20.71± 0.36 ab	35.71 ± 0.36 b
2	2.75± 0.32 a	1.23 ± 0.16 a	100.5 ± 0.55 a	27.5± 0.38 a	25 ± 0.29 c

*Means followed by the same letter within a column are not significantly different at p < 0.05 according to Tukey’s post hoc test. All values are stated as mean ± SD.

These results indicate that moderate concentrations stimulate both shoot and callus formation, while higher concentrations strongly favor shoot proliferation over callus initiation.

### 3.3 Effect of *C. myrrha* gum-resins ethanolic extract (g·L^-1^) concentrations on chlorophyll a, b, total a+b and carotenoid contents of *A. fragrantissima*

**Figure 2:**
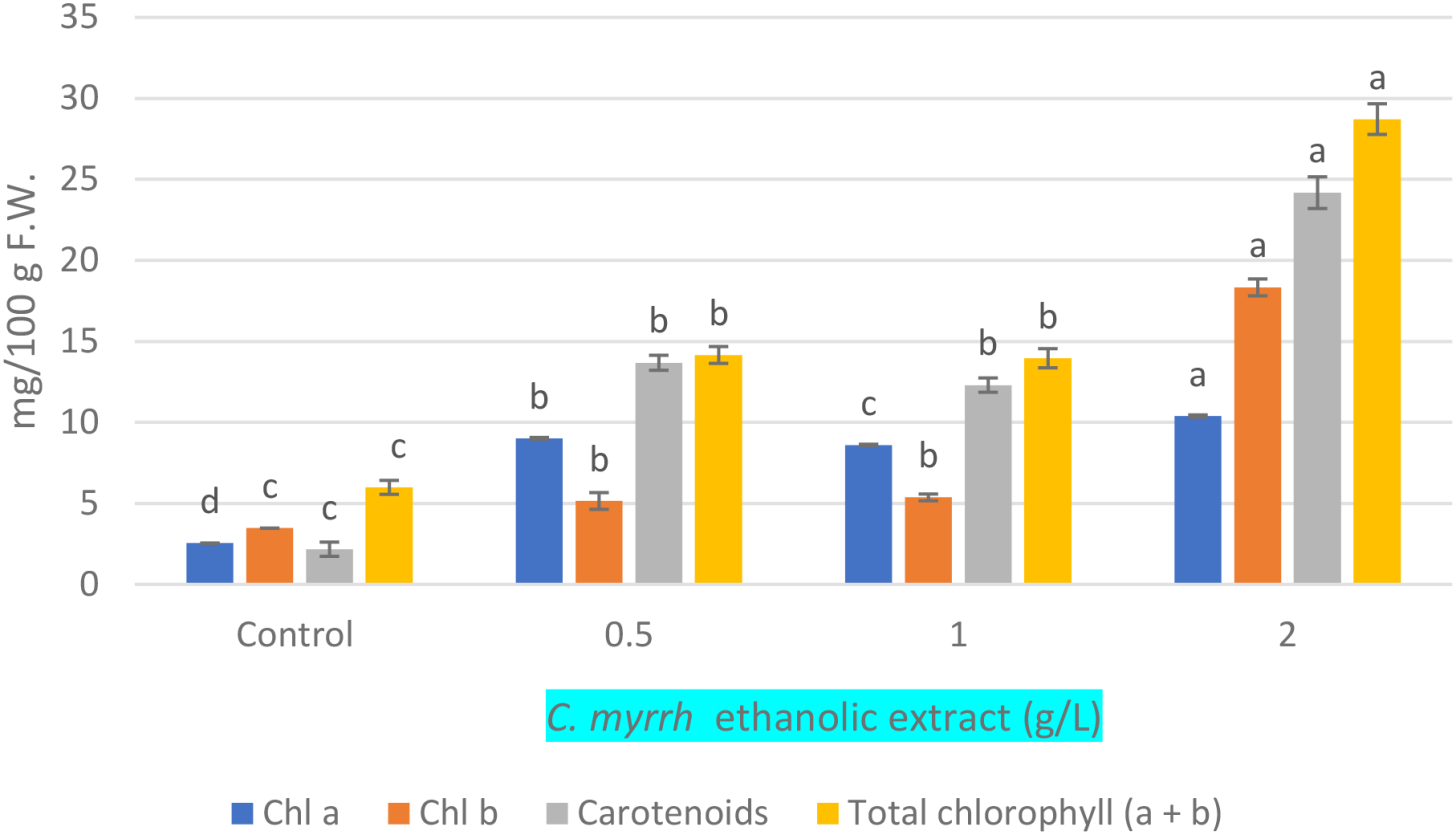
Effect of *C. myrrha* gum-resin ethanolic extract (g·L^-1^) concentrations on chlorophyll (a, b) total chlorophyll (a+b), and carotenoid contents of *A. fragrantissima*.

**Figure 2** shows that *C. myrrha* ethanolic extract significantly increased photosynthetic pigment levels in *A. fragrantissima*. Chlorophyll a, chlorophyll b, total chlorophyll (a + b), and carotenoids all increased significantly with rising extract concentration, with the 2.0 g·L^-1^ treatment yielding the highest values for all pigments.

### 3.4 Callus induction from root explants: percentage and biomass

**Figure 3:**
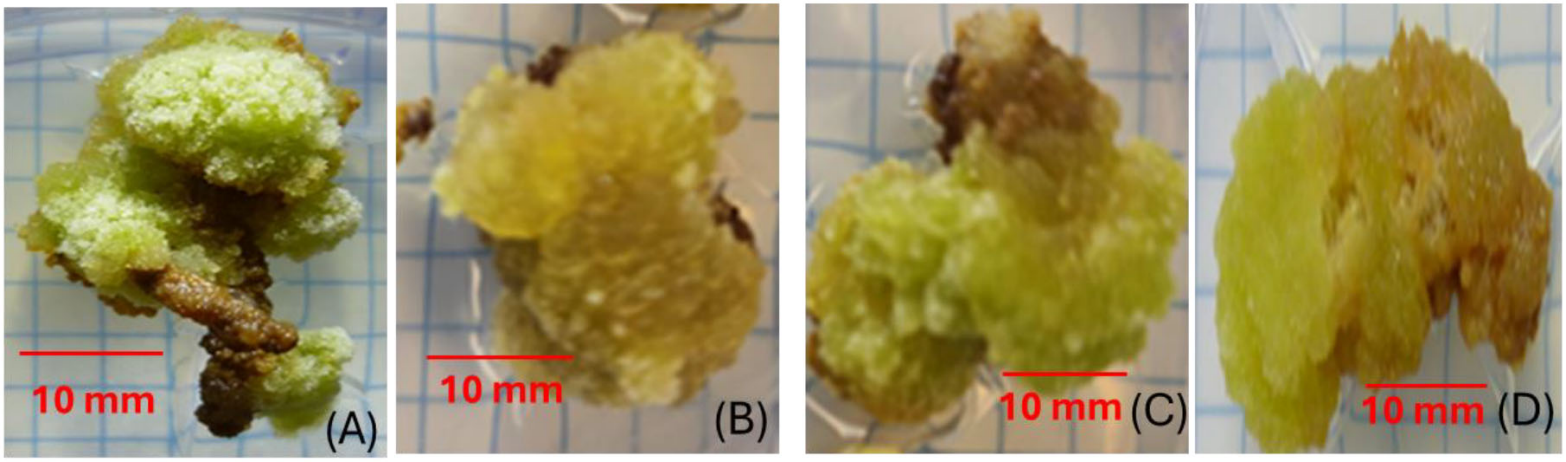
Effect of *C*. *myrrha* gum-resin ethanolic extract concentrations on callus induction from *A*. *fragrantissima* root segments. **(A)** Control, **(B)** 0.5 g·L^-1^, **(C)** 1.0 g·L^-1^, and **(D)** 2.0 g·L^-1^ are concentrations of *C*. *myrrha* gum-resin ethanolic extract.

**Figure 4:**
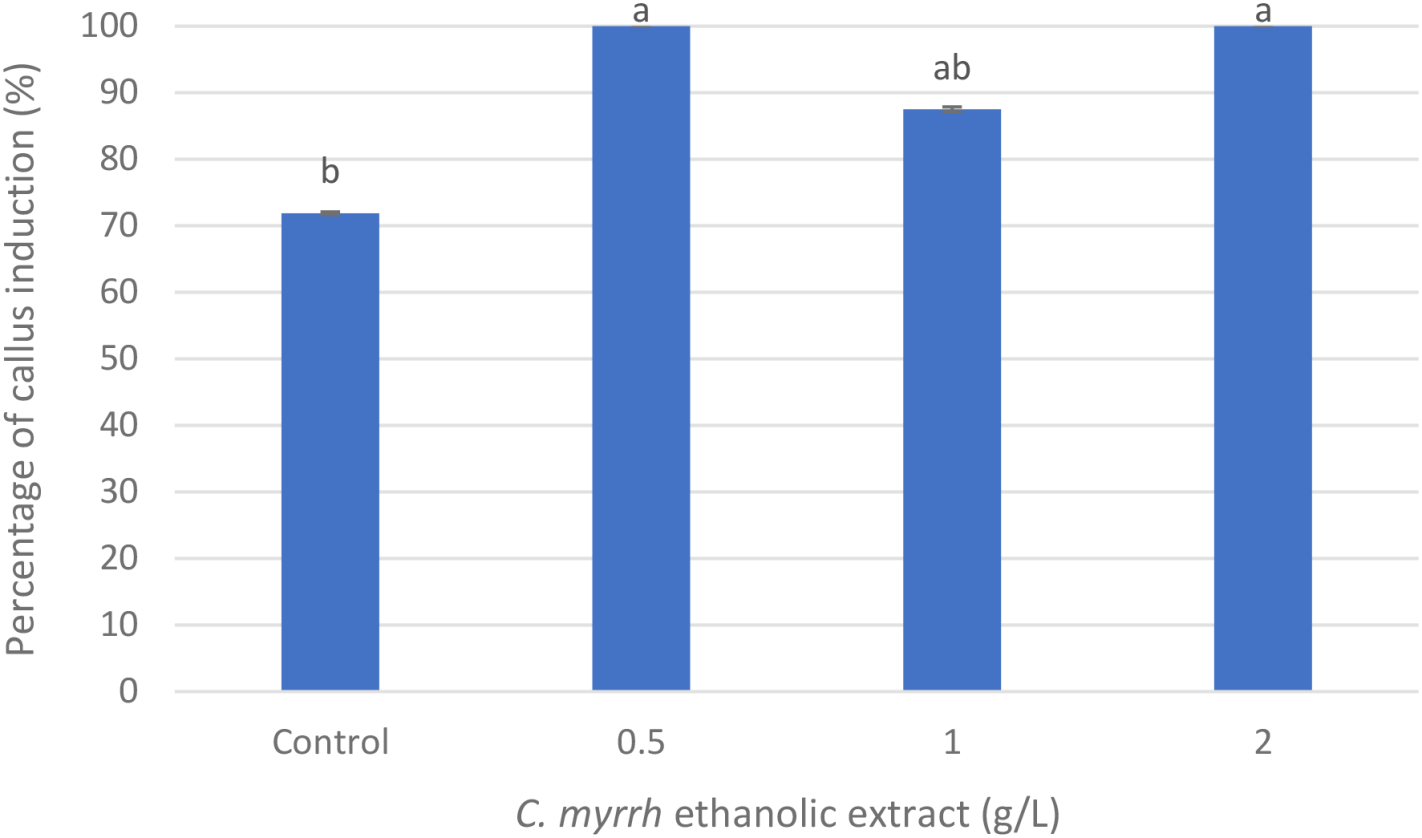
Effect of *C. myrrha* gum-resin ethanolic extract (g·L^-1^) concentrations on callus induction (%) from root segments of *A. fragrantissima*. Callus percentage values represent the mean of 12 replicates (Petri dishes) per treatment.

**Figure 5:**
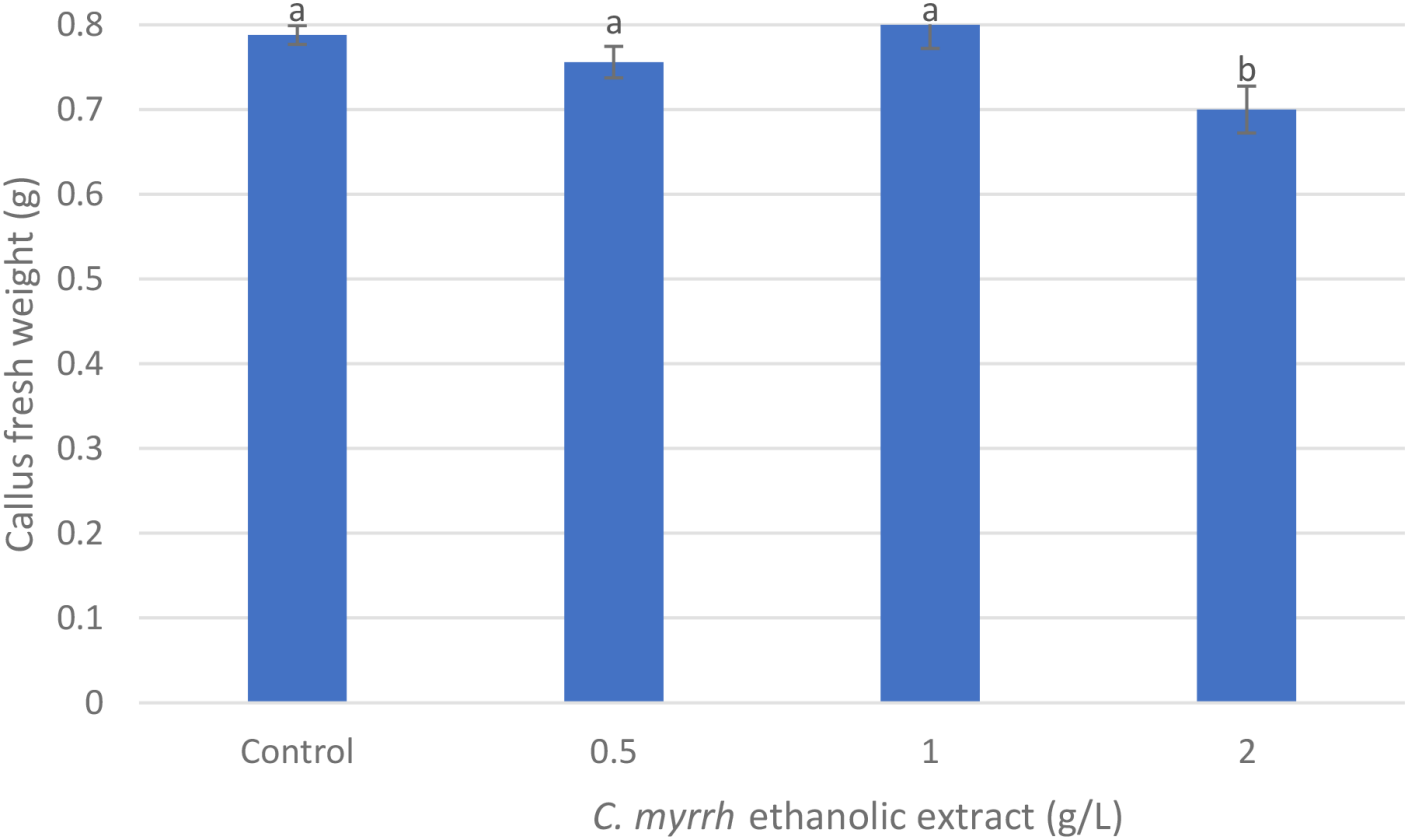
Effect of *C. myrrha* gum-resin ethanolic extract (g·L^-1^) concentrations on callus fresh weight (g) from root segments of *A. fragrantissima*. Callus fresh weight values represent the mean of 12 replicates (Petri dishes) per treatment.

The ethanolic extract of *C. myrrha* gum-resin had a substantial impact on callus induction in *A. fragrantissima*. Callus formation reached 100% at both 0.5 g/L and 2 g/L *C. myrrha* gum-resin ethanolic concentrations (**Figures 3B, D**, **4**), compared with 71.88% in the control and 87.5% at 1.0 g·L^-1^ (**Figures 3A, C**, **4**). However, the fresh weight of the resulting callus did not follow the same trend (**Figures 3**, **5**). The control, 0.5 g·L^-1^, and 1.0 g·L^-1^*C. myrrha* gum-resin ethanolic extract treatments produced comparable biomass values of approximately 0.75–0.80 g (**Figure 5**). In contrast, the 2 g/L *C. myrrha* gum-resin ethanolic extract treatment generated a significantly lower fresh weight of ~0.70 g (**Figure 5**). According to these findings, the biomass accumulation in the callus may be decreased at the maximum concentration tested, even while larger concentrations of *C. myrrha* gum-resin ethanolic extract may encourage a higher proportion of callus development (**Figures 3**-**5**).

### 3.5 Effect of *C. myrrha* ethanolic extract on the phytochemical composition of *in vitro A. fragrantissima* shoot tip plantlets

Different quantities of the ethanolic extract of C. myrrha gum-resins had a substantial impact on the composition of the methanolic extract isolated from shoot tip explants of A. fragrantissima plantlets (**Table 3**, **Supplementary Table S1**). The predominant compound in all treatments was desulphosinigrin, which had the largest phytochemical compounds area percentage at 1.0 g·L^-1^ (18.58%), followed by the control (15.57%), 2.0 g·L^-1^ (12.32%), and the lowest at 0.5 g/L (0.88%). Several phytochemical compounds were upregulated or newly induced in A. fragrantissima in response to the various C. myrrha treatment concentrations. Interestingly, at 2.0 g·L^-1^ (11.52%) and 1.0 g·L^-1^ (10.74%), the oleic acid isomer (9-octadecenoic acid) significantly increased in comparison to the control (1.34%). The trimethylsilyl ester derivative palmitic acid, which peaked at 0.5 g·L^-1^ (7.8%), was present in all treated samples but was not present in the control. Significant increases were also observed in treated samples for compounds such 2,2-dimethyl-5-[2-(2-trimethylsilylethoxymethoxy)-propyl]-[1,3]dioxolane-4-carboxaldehyde and 17-octade cynoic acid, especially at 1 and 2.0 g·L^-1^. Compounds that were absent from the control but present in one or more of the treated groups included cyclopentanetridecanoic acid methyl ester, 1H-indol-5-ol derivatives, and mannopyranoside derivatives. During *in-vitro* propagation, these results imply that the ethanolic extract of C. myrrha gum-resin not only modifies but also may improve the production of important phytochemical compounds constituents in A. fragrantissima (**Table 3**).

**Table 3:** Effect of *C. myrrha* gum-resin ethanolic extract (g·L^-1^) on the phytochemical compounds composition of *A. fragrantissima* during the *in-vitro* plantlet multiplication stage.

Phytochemical compounds name	Area%			
	*C. myrrha* gum -resin ethanolic extract (g·L^-1^)			
	Control	0.5	1.0	2.0
Desulphosinigrin	15.57b*	0.88d	18.58a	12.32c
2-Aminoethanethiol hydrogen sulfate (ester)	2.8a	0.73c	–	1.49b
Hexadecanoic acid, 2,3-dihydroxypropyl ester (Glyceryl palmitate)	1.73a	0.5c	1.05b	1.18b
Pentadecanoic acid	1.49b	1.17c	1.81a	0.78d
9-Octadecenoic acid (oleic acid isomer))	1.34c	0.39d	10.74b	11.52a
Palmitic Acid, trimethylsilyl ester derivative	–	7.8a	3.45c	4.52b
Oleic acid	1.27b	1.25b	1.26b	1.87a
17-Octadecynoic acid	0.65c	3.66a	3.64a	3.37b
2,2-Dimethyl-5-[2-(2-trimethylsilylethoxymethoxy)-propyl]-[1,3]dioxolane-4-carboxaldehyde	–	5.73b	10.87a	10.99a
9,12,15-Octadecatrienoic acid, trimethylsilyl ester derivative	–	0.44c	2.35a	0.88b
Cyclopentanetridecanoic acid, methyl ester	–	–	1.9a	0.86b
1,3,5-Triazine-2,4-diamine, 6-chloro-N-ethyl-	0.83b	–	0.67c	1.61a
1H-Indol-5-ol, 3-(2-aminoethyl)-	–	0.95b	0.94b	1.04a
α-D-Mannopyranoside, methyl, cyclic 2,3:4,6-bis(butylboronate)	–	0.8b	–	1.51a
2-Trimethylsiloxy-6-hexadecenoic acid, methyl ester	–	1.51a	1.63a	1.4b

Means followed by the same letter within a column are not significantly different at p < 0.05 according to Tukey’s post hoc test.

### 3.6 GC-MS analysis of methanolic extracts from *A. fragrantissima* callus-stage plantlets treated with *C. myrrha* gum-resin extract (g·L^-1^)

The effect of *C. myrrha* gum-resin ethanolic extract on selected metabolites in A. fragrantissima *in vitro* plantlets is presented in **Table 4**. Thymidine content was lowest at 0.5 g·L^-1^ (0.46%) and in the control (2.14%), reached its maximum at 1.0 g·L^-1^ (4.48%), and was undetectable at 2.0 g·L^-1^. Similarly, 4H-pyran-4-one, 2,3-dihydro-3,5-dihydroxy-6-methyl- increased with extract concentration, rising from 7.07% in the control to 10.16% at 1.0 g·L^-1^, before decreasing to 6.59% at 2.0 g·L^-1^. When compared to the control and other treatments, desulphosinigrin showed a notable increase at 1.0 g·L^-1^ (12.07%). The control had the highest oleic acid concentration (3.41%), which increased little at 2.0 g·L^-1^ (2.29%), but considerably at 0.5 and 1.0 g·L^-1^. Both 9-octadecenoic acid and 11-octadecenoic acid methyl esters exhibited different reactions; 9-octadecenoic acid remained largely unchanged during treatments, while 11-octadecenoic acid methyl ester was only found in the control and 0.5 g·L^-1^ samples.

**Table 4:** Effect of *C. myrrha* gum-resin ethanolic extract (g·L^-1^) concentrations on the compositions of phytochemical composition of methanol extracts prepared from *A. fragrantissima in-vitro* plantlets callus stage. Methanol extracts, and the phytochemical composition were analyzed by GC-MS. Three samples were collected from each treatment for analysis.

Phytochemical compounds name	Area%			
	*C. myrrha* gum -resins ethanolic extract (g·L^-1^)			
	Control	0.5	1.0	2.0
Thymidine	2.14 b	0.46 c	4.48 a	–
4H-Pyran-4-one, 2,3-dihydro-3,5-dihydroxy-6-methyl-	7.07c	8.82b	10.16a	6.59c
Melezitose	2.69a	0.63d	1.07c	1.3b
Desulphosinigrin	2.5b	2.51b	12.07a	1.33c
Oleic Acid	3.41a	0.44c	0.21d	2.29b
9-Octadecenoic acid, methyl ester	2.57a	2.47a	1.38b	2.47a
11-Octadecenoic acid, methyl ester	0.80 b	2.42a	–	–
Palmitic Acid	6.33a	4.8b	2.1c	6.86a
Hexadecanoic Acid	11.63a	12.24a	9.32b	9.25b

*Means followed by the same letter within a column are not significantly different at p < 0.05 according to Tukey’s post hoc test.

The levels of palmitic acid were lowest at 0.5 and 1.0 g·L^-1^ and greatest at 2.0 g·L^-1^ (6.86%) and the control (6.33%). Likewise, the control and 0.5 g·L^-1^ treatments had the highest levels of hexadecanoic acid (11.63% and 12.24%), but at higher doses, they dropped to about 9.3%. These findings suggest that the ethanolic extract of *C. myrrha* gum-resin regulates the synthesis of important fatty acid derivatives and secondary metabolites in callus tissues, with notable increases in particular bioactive compounds, particularly following a 1.0 g·L^-1^ treatment.

### 3.7 GC-MS analysis of methanolic extracts from *A. fragrantissima* callus and shoot tip plantlets treated with *C. myrrha* gum-resin extract

Under the influence of C. myrrha gum-resin ethanolic extract, the methanolic extract content of A. fragrantissima tissues differed significantly between callus and shoot tip tissues, according to data in **Table 5**. Across most concentrations, desulphosinigrin compound was consistently higher in the explants produced from shoot tips, particularly at 1.0 g·L^-1^ and 2.0 g·L^-1^, suggesting that the tissues of the shoot tips as explant encourage its accumulation.

**Table 5:** Comparative summary of GC–MS-identified phytochemical compounds in methanolic extracts of *A. fragrantissima in-vitro* plantlets, highlighting differences between callus and shoot tip tissues following treatment with *C. myrrha* gum-resin ethanolic extract (g·L^-1^).

Phytochemical compounds name	*C. myrrha* gum -resins ethanolic extract (g·L^-1^)	Area%		Higher in
		Callus explant	Shoot tip explant	
Desulphosinigrin	Control	2.5	15.57	Shoot tip
	0.5	2.51	0.88	Callus
	1.0	12.07	18.58	Shoot tip
	2.0	1.33	12.32	Shoot tip
Oleic Acid	Control	3.41	1.27	Callus
	0.5	0.44	1.25	Shoot tip
	1.0	0.21	1.26	Shoot tip
	2.0	2.29	1.87	Callus
9-Octadecenoic acid (Z)-, methyl ester	Control	2.57	1.34	Callus
	0.5	2.47	0.39	Callus
	1.0	1.38	10.74	Shoot tip
	2.0	2.47	11.52	Shoot tip
Palmitic Acid/Hexadecanoic Acid	Control	6.33/11.63	–/–	Callus
	0.5	4.80/12.24	7.80/–	Mixed (Callus >)
	1.0	2.10/9.32	3.45/–	Mixed (Callus >)
	2.0	6.86/9.25	4.52/–	Callus
2-Trimethylsiloxy-6-hexadecenoic acid, methyl ester	Control	–	–	–
	0.5	–	1.51	Shoot tip
	1.0	–	1.63	Shoot tip
	2.0	–	1.4	Shoot tip
1H-Indol-5-ol, 3-(2-aminoethyl)-	Control	–	–	–
	0.5	–	0.95	Shoot tip
	1.0	–	0.94	Shoot tip
	2.0	–	1.04	Shoot tip

Callus and shoot tip tissues of *A. fragrantissima* showed distinct responses to *C. myrrha* gum-resin ethanolic extract. Callus tissue generally had higher levels of oleic acid, especially in the control (3.41%) and at 2.0 g·L^-1^ (2.29%), while shoot tips accumulated more at lower concentrations (0.5–1.0 g·L^-1^). Similarly, 9-octadecenoic acid (Z)-methyl ester was more abundant in callus at low concentrations but dominated in shoot tips at 1.0 and 2.0 g·L^-1^ (10.74% and 11.52%, respectively). Palmitic acid and hexadecanoic acid were consistently higher in callus, whereas shoot tips had little or no production of these fatty acids. Some compounds, such as 2-trimethylsiloxy-6-hexadecenoic acid methyl ester and 1H-indol-5-ol, 3-(2-aminoethyl)-, were only found in shoot tips, indicating tissue-specific induction of certain secondary metabolites. Overall, callus favored fatty acid accumulation, while shoot tips showed higher levels of bioactive and aromatic compounds, highlighting functional differences in metabolic responses to *C. myrrha* gum-resin ethanolic extract.

## 4 Discussion

### 4.1 Phytochemical analysis of *C. myrrha* gum-resin ethanolic extract using GC–MS

The GC–MS profile of *C. myrrha* gum-resin ethanolic extract revealed a chemically diverse mixture of mono- and sesquiterpenes, oxygenated derivatives, and minor aromatics. Such terpenoid-rich systems are frequently involved in plant defense signaling, secondary-metabolite activation, and hormonal cross-talk (Liu et al., 2022; Câmara et al., 2024). Sesquiterpenes like caryophyllene, caryophyllene oxide, humulene, and valencene are well-documented modulators of plant–microbe interactions and defense cascades, with the ability to upregulate pathogenesis-related proteins, MAPK cascades, and phenylpropanoid enzymes (Hilgers et al., 2021; Ogundajo et al., 2021; Gao et al., 2022; Song et al., 2024; Xu et al., 2024) Additionally, menthofuran has been linked to oxidative-stress modulation and detoxification pathways through stimulation of antioxidant enzymes, including superoxide dismutase and catalase (Alves et al., 2023; Jariani et al., 2024). Similarly, phenolic constituents such as 3,5-xylenol may stimulate the activity of key defense enzymes, including phenylalanine ammonia-lyase (PAL) and peroxidases that are directly linked to the biosynthesis of secondary metabolites (Kisiel et al., 2024). These observations support the idea that the resin constituents act as elicitors when introduced into *in-vitro* culture.

### 4.2 Impact of varying concentrations of *C. myrrha* Gum-resin ethanolic extract on growth and photosynthetic pigments

*C. myrrha* gum-resin ethanolic extract significantly accelerated *A. fragrantissima* growth during the multiplication stage. Plant tissues have higher concentrations of photosynthetic pigments in concert with this growth stimulation. Furthermore, the extract significantly enhanced callus formation when applied to root explants, increasing both the callus fresh weight and the callus induction percentage. These findings suggest that *C. myrrha* gum-resin extract functions as a promising natural biostimulant. Consistent with these results, several studies have reported that plant-derived extracts can effectively enhance plant growth, development, and *in-vitro* callus formation (Markin et al., 2023; David Raja et al., 2025; El Sherif et al., 2025). The growth-promoting effects documented in this study—including increased shoot number, higher fresh biomass, and elevated pigment levels—are consistent with the biostimulant role of terpenoids and their derivatives. The observed increase in photosynthetic pigments suggests an enhancement in photosynthetic efficiency, which is often associated with improved carbon assimilation and energy availability for secondary metabolism (Tao et al., 2023). Increased pigment levels are known to support the biosynthesis of stress-related metabolites (Ren et al., 2017), including terpenes and phenolics, which help mitigate oxidative damage by scavenging reactive oxygen species and stabilizing cellular structures (Kurhaluk et al., 2025).

### 4.3 Role of specific bioactive compounds

Compounds such as limonene and myrcenol are known to influence nutrient availability, membrane permeability, and energy metabolism, thereby enhancing cell division and organogenesis via hormone-like or signaling pathways (Hsiung et al., 2013; Yang et al., 2024; Liu et al., 2025). Likewise, sesquiterpenes like caryophyllene, valencene and germacrene B may interact with auxin signaling, further supporting cell proliferation and tissue differentiation (Pazouki et al., 2015; Yin et al., 2022; Song et al., 2024) α-Terpinene, β-pinene, and azulene derivatives act primarily as antioxidants, reducing reactive oxygen species (ROS) in meristematic zones, thereby safeguarding DNA integrity during cell division and maintaining high mitotic rates under stress (Muir and Hansch, 1961; Boncan et al., 2020; Tian et al., 2020; Menezes et al., 2021).

### 4.4 Modulation of phytochemical composition

By increasing the accumulation of metabolites linked to growth and development, the addition of *C. myrrha* gum-resin ethanolic extract had a substantial impact on the phytochemical compounds content of *A. fragrantissima* in both callus tissues and shoot tip explants. Across all treatments, desulphosinigrin was the most common chemical, with the highest concentration observed in callus tissue and shoot tip explants treated with *C. myrrha* gum-resin ethanolic extract. This glucosinolate is recognized for its anticancer and antimicrobial properties, suggesting that elicitor treatments may enhance the plant’s therapeutic potential (Youssef et al., 2023). In shoot tip explants, the marked increase in oleic acid (9-octadecenoic acid) at higher extract concentrations suggests enhanced lipid biosynthesis, which is potentially for infectious diseases such as coronavirus disease (COVID-19) (Arundina et al., 2024). The induction of palmitic acid, which was absent in the control but present in the treated samples, indicates stimulation of fatty acid metabolism, critical for maintaining membrane structural integrity and energy storage. While hexadecanoic acid levels were highest in the control and 0.5 g·L^-1^ treatments, suggesting a concentration-dependent biosynthesis in response to the extract (El Sherif et al., 2025). In callus tissues, thymidine content peaked at 1.0 g·L^-1^ but was undetectable at 2.0 g·L^-1^, indicating that higher concentrations may inhibit its biosynthesis (El Sherif et al., 2020; Al Dayel and El Sherif, 2022; El Sherif et al., 2025). Thymidine is essential for DNA synthesis and repair, highlighting the importance of optimal dosing in elicitor treatments (Jeong et al., 2021). The compound 4H-pyran-4-one, 2,3-dihydro-3,5-dihydroxy-6-methyl-, increased with extract concentration, reaching a maximum of 10.16% at 1.0 g·L^-1^. This compound has been associated with antioxidant activity, which can help mitigate oxidative stress and reduce the risk of chronic diseases in humans (Olaniyan et al., 2018; Sato et al., 2025).

### 4.5 Plant extract–based biostimulants as sustainable enhancers of growth and bioactive compounds in tissue culture

Plant extract-based biostimulants are playing an increasingly important and powerful role in plant tissue culture by enhancing morphogenesis, promoting stress tolerance, improving phytochemical content, and providing eco-friendly alternatives to synthetic growth regulators. Their complex composition offers multi-target benefits, making them ideal for enhancing micropropagation efficiency, particularly in medicinal and stress-sensitive plant species (Göre, 2024; Marciniak and Sochacki, 2025). The findings of this study provide important insight into the potential use of plant extract-based biostimulants in plant tissue culture systems. The ability of *C. myrrha* extract to enhance shoot proliferation, pigment biosynthesis, and phytochemical accumulation in *A. fragrantissima* demonstrates that natural plant-derived compounds can act as effective elicitor of *in-vitro* growth and metabolism (Razzaq et al., 2025). Unlike conventional synthetic growth regulators, plant extracts contain a complex mixture of secondary metabolites such as terpenoids, phenolics, and antioxidant compounds that may act synergistically to stimulate cellular division, differentiation, and metabolic activity (Al Nooh et al., 2025; Jamiołkowska, 2020). Terpenoids not only participate in plant defense and ecological interactions, but also help regulate cell growth, elongation, and membrane stability, and may provide photoprotection by interacting with reactive oxygen species produced during stress conditions, thereby supporting overall growth and resilience in plants (Khan et al., 2025). Phenolic compounds play important roles in physiological processes such as cell division, photosynthesis, and nutrient mobilization; they can also act as antioxidants by scavenging harmful ROS and protecting cell structures under stress, while influencing hormone signaling and developmental pathways that contribute to root and shoot differentiation (Kumar et al., 2023)Antioxidants in plant extracts, which include phenolics and other secondary metabolites, help maintain cellular integrity by neutralizing oxidative stress that can disrupt metabolic activity, thereby preserving enzyme function and supporting physiological processes essential for growth and stress tolerance (Rao and Zheng, 2025). These compounds enhance nutrient uptake, metabolic efficiency, and stress resilience, ultimately improving plant growth, vigor, and the accumulation of bioactive compounds in plant tissues.

The concentration-dependent improvements observed in this study highlight how plant extract–based biostimulants can be optimized to support specific developmental processes, offering a sustainable approach to improving micropropagation efficiency and plantlet quality (Miler et al., 2024). This research offers insight into the application of plant extract–based biostimulants in tissue culture, demonstrating how plant-derived extracts can mimic stress or hormonal signals to promote growth, enhance shoot proliferation, and increase the accumulation of valuable bioactive compounds. It highlights biostimulants as sustainable alternatives or supplements to conventional plant growth regulators, offering practical strategies to improve tissue culture efficiency and metabolite production.

### 4.5 Tissue-specific responses

A comparative analysis between shoot tip explants and callus tissues revealed tissue-specific responses to the ethanolic extract. Shoot tip explants favored the accumulation of specific bioactive and aromatic compounds, while callus tissues exhibited a stronger biosynthesis of fatty acid-related constituents. This functional divergence underscores the influence of tissue type on metabolic responses to elicitor treatments. Future work should include mechanistic studies, such as enzyme assays and transcriptomic analyses, to clarify how extract constituents interact with biosynthetic pathways. It would also be valuable to test combined treatments of *C. myrrha* gum-resin ethanolic extract with classical plant growth regulators to explore potential synergistic effects. Such refinements could lead to optimized *in-vitro* production systems for metabolite-rich tissues of *A. fragrantissima* and other medicinally important or endangered species.

## 5 Conclusions

Ethanolic extracts of *C. myrrha* gum-resin act efficiently as a natural biostimulant and elicitor in *A. fragrantissima* plants grown under tissue culture conditions. The extract enhanced shoot proliferation, increased the content of photosynthetic pigments, and stimulated callus formation. It also significantly altered the metabolite profile, producing tissue-specific effects. Shoot tips accumulated a broader range of aromatic and therapeutic metabolites, such as desulphosinigrin and thymidine, while callus tissue was enriched in fatty acid derivatives, including palmitic and hexadecanoic acids. Many of the elicited compounds, including desulphosinigrin, thymidine, and 4H-pyran-4-one derivatives, possess recognized anticancer, antimicrobial, and antioxidant properties. Overall, *C. myrrha* gum-resin extract represents a sustainable and practical agent for enhancing the production of valuable secondary metabolites, supporting both the conservation and scalable *in vitro* propagation of this medicinal plant.

## Data Availability

The original contributions presented in the study are included in the article/**Supplementary Material**. Further inquiries can be directed to the corresponding author.

## Supplementary material

Supplementary Table 1 Effect of *Commiphora myrrha* gum -resins ethanolic extract (g·L^-1^) concentrations on the compositions of phytochemical compounds prepared from *A. fragrantissima in-vitro* plantlets multiplication stage.

Supplementary Table 2 Effect of *Commiphora myrrha* gum -resins ethanolic extract (g·L^-1^) concentrations on the compositions of phytochemical compounds prepared from *A. fragrantissima in-vitro* plantlets callus stage.
