# Supplementary material for: Ethanolic extract of Commiphora myrrha gum-resin promotes therapeutic compound accumulation in Achillea fragrantissima under in vitro culture
Source: Front Plant Sci. 2026 Jan 28;16:1714322. doi: 10.3389/fpls.2025.1714322 (PMC12891227; doi:10.3389/fpls.2025.1714322)
Supplement: Supplementary Table 1 — Effect of Commiphora myrrha gum -resins ethanolic extract (g·L-1) concentrations on the compositions of phytochemical compounds prepared from A. fragrantissima in-vitro plantlets multiplication stage. [file Table1.docx]

**Sup. Table S1. Effect of *Commiphora myrrha* gum -resins ethanolic extract (g/L) concentrations on the compositions of** **Phytochemical compounds prepared from *A. fragrantissima* in-vitro plantlets multiplication stage.**

| *C. myrrha* (g/L) | RT | **Phytochemical compounds** Name | Area % | Molecular Formula | | Molecular Weight |
| --- | --- | --- | --- | --- | --- | --- |
| Control | 16.56 | 2-aminoethanethiol hydrogen sulfate (ester) | 2.80 | C2H7NO3S2 | | 157 |
|  | 19.15 | pentadecanoic acid | 1.49 | C15H30O2 | | 242 |
|  | 20.67 | Desulphosinigrin | 15.57 | C10H17NO6S | | 279 |
|  | 22.77 | Hexadecanoic acid, 2,3-dihydroxypropyl ester | 1.73 | C19H38O4 | | 330 |
|  | 23.40 | Tetradecanoic acid | 3.12 | C14H28O2 | | 228 |
|  | 24.60 | 1,3,5-triazine-2,4-diamine, 6-chloro-N-ethyl- | 0.83 | C5H8ClN5 | | 173 |
|  | 24.88 | α-D-glucopyranoside, methyl 2,3-bis-O-(trimethylsilyl)-, cyclic butylboronate | 0.71 | C17H37BO6Si2 | | 404 |
|  | 26.23 | Cyclopropanebutanoic acid, 2-2-pentylcyclopropyl)methyl]cyclopropyl]methyl]cyclopropyl]methyl]-, methyl ester | 2.98 | C25H42O2 | | 374 |
|  | 27.39 | n-Hexadecanoic acid | 19.81 | C16H32O2 | | 256 |
|  | 28.73 | 17-Octadecynoic acid, TMS derivative | 0.76 | C21H40O2Si | | 352 |
|  | 29.28 | 9- Octadecenoic acid | 1.34 | C18H34O2 | | 282 |
|  | 29.45 | 9-Octadecenoic acid -, methyl ester | 1.77 | C19H36O2 | | 296 |
|  | 30.61 | cis-Vaccenic acid | 39.36 | C18H34O2 | | 282 |
|  | 31.03 | Oleic Acid | 1.27 | C18H34O2 | | 282 |
|  | 32.76 | Glycidyl palmitate | 2.18 | C19H36O3 | | 312 |
|  | 35.59 | Glycidyl oleate | 2.78 | C21H38O3 | | 338 |
|  | 36.08 | 9,12-Octadecadienoic acid | 0.86 | C18H32O2 | | 280 |
|  | 37.98 | 17-Octadecynoic acid | 0.65 | C18H32O2 | | 280 |
|  | | | | | | |
| 0.5 | 7.29 | Benzeneethanamine, N,α,α-trimethyl | 0.41 | C11H17N | | 163 |
|  | 7.41 | 2-Trimethylsiloxy-6-hexadecenoic acid, methyl ester | 1.51 | C20H40O3Si | | 356 |
|  | 7.61 | Melezitose | 0.73 | C18H32O16 | | 504 |
|  | 8.53 | 2-Propyl-tetrahydropyran-3-ol | 3.21 | C8H16O2 | | 144 |
|  | 10.12 | α-D-mannopyranoside, methyl, cyclic 2,3:4,6-bis(butylboronate | 0.80 | C15H28B2O6 | | 326 |
|  | 10.19 | α-D-galactopyranoside, methyl 2,6-bis-O-(trimethylsilyl)-, cyclic butylboronate | 0.68 | C17H37BO6Si2 | | 404 |
|  | 11.53 | trans-2-undecenoic acid | 1.23 | C11H20O2 | | 184 |
|  | 12.22 | Spiro[5.5]undecane-1,7-dione | 1.07 | C11H16O2 | | 180 |
|  | 12.78 | 9,12,15-octadecatrienoic acid, 2-[(trimethylsilyl)oxy]-1-[[(trimethylsilyl)oxy]methyl]ethyl ester, | 0.44 | C27H52O4Si2 | | 496 |
|  | 12.83 | 5,8,11-Eicosatrienoic acid, (Z)-, TMS derivative | 0.37 | C23H42O2Si | | 378 |
|  | 13.13 | 5-Dimethyl(trimethylsilyl)silyloxytridecane | 1.71 | C18H42OSi2 | | 330 |
|  | 14.61 | 2,2-Dimethyl-5-[2-(2-trimethylsilylethoxymethoxy)-propyl]-[1,3]dioxolane-4-carboxaldehyde | 2.35 | C15H30O5Si | | 318 |
|  | 16.16 | Octadecanoic acid, 9,10-epoxy-18-(trimethylsiloxy)-, methyl ester, cis- | 0.75 | C22H44O4Si | | 400 |
|  | 16.56 | trans-2-Dodecenoic acid | 0.69 | C12H22O2 | | 198 |
|  | 16.60 | 2-aminoethanethiol hydrogen sulfate (ester) | 0.73 | C2H7NO3S2 | | 157 |
|  | 16.71 | 1H-indol-5-ol, 3-(2-aminoethyl)- | 0.95 | C10H12N2O | | 176 |
|  | 17.36 | 2-Hydroxyisocaproic acid, TMS derivative | 12.22 | C9H20O3Si | | 204 |
|  | 18.11 | Dodecanoic acid, 2,3-bis(acetyloxy)propyl ester | 0.79 | C19H34O6 | | 358 |
|  | 18.65 | Tristrimethylsilyl ether derivative of 1,25-dihydroxyvitamin D2 | 0.55 | C37H68O3Si3 | | 644 |
|  | 19.35 | à-D-Galactopyranose, 6-O-(trimethylsilyl)-, cyclic 1,2:3,4-bis(methylboronate) | 0.67 | C11H22B2O6Si | | 300 |
|  | 19.53 | Benzeneethanamine, N,α,α-trimethyl | 0.47 | C11H17N |  | 163 |
|  | 20.73 | Ethyl à-d-glucopyranoside | 5.78 | C8H16O6 |  | 208 |
|  | 21.04 | Anastrozole | 8.70 | C17H19N5 | | 293 |
|  | 22.18 | 2,2-Dimethyl-5-[2-(2-trimethylsilylethoxymethoxy)-propyl]-[1,3]dioxolane-4-carboxaldehyde | 5.73 | C15H30O5Si | | 318 |
|  | 22.32 | 17-Octadecynoic acid, TMS derivative | 6.68 | C21H40O2Si | | 352 |
|  | 22.67 | Octadecanoic acid, 9,10-dichloro-, methyl ester | 0.56 | C19H36Cl2O2 | | 366 |
|  | 22.95 | Hexadecanoic acid, 2,3-dihydroxypropyl ester | 0.50 | C19H38O4 | | 330 |
|  | 24.04 | Desulphosinigrin | 0.88 | C10H17NO6S | | 279 |
|  | 24.86 | PENTADECANOIC ACID | 1.17 | C15H30O2 | | 242 |
|  | 25.19 | [1,1'-Bicyclopropyl]-2-octanoic acid, 2'-hexyl-, methyl ester | 1.72 | C21H38O2 | | 322 |
|  | 25.50 | Myristic acid, TMS derivative | 1.05 | C17H36O2Si | | 300 |
|  | 25.93 | Dodecanoic acid, 3-hydroxy- | 0.60 | C12H24O3 | | 216 |
|  | 26.67 | 17-Octadecynoic acid | 3.66 | C18H32O2 | | 280 |
|  | 26.81 | Pentadecanoic acid, 14-methyl-, methyl ester | 2.60 | C17H34O2 | | 270 |
|  | 27.45 | Octadecanoic acid, 9,10-epoxy-18-(trimethylsiloxy)-, methyl ester, cis- | 0.52 | C22H44O4Si | | 400 |
|  | 27.70 | n-Hexadecanoic acid | 6.64 | C16H32O2 | | 256 |
|  | 29.32 | Palmitic Acid, TMS derivative | 7.80 | C19H40O2Si | | 328 |
|  | 29.83 | Oleic Acid | 1.25 | C18H34O2 | | 282 |
|  | 30.03 | 11-Octadecenoic acid, methyl ester | 2.55 | C19H36O2 | | 296 |
|  | 30.18 | 2-aminoethanethiol hydrogen sulfate (ester) | 0.60 | C2H7NO3S2 | | 157 |
|  | 30.59 | Cyclopropanepentanoic acid, 2-undecyl-, methyl ester, trans- | 0.70 | C20H38O2 | | 310 |
|  | 30.80 | **9-octadecenoic acid (Z)-** | 0.39 | C18H34O2 | | 282 |
|  | 30.88 | 9-Hexadecenoic acid | 1.98 | C16H30O2 | | 254 |
|  | 32.31 | Oleic acid, trimethylsilyl ester | 1.07 | C21H42O2Si | | 354 |
|  | 32.84 | Trimethylsilyl (9E)-9-octadecenoate | 0.89 | C21H42O2Si | | 354 |
|  | 33.24 | 1,25-Dihydroxyvitamin D3, TMS derivative | 0.64 | C30H52O3Si | | 488 |
|  | 36.69 | 1-hexadecanol, 2-methyl- | 2.03 | C17H36O |  | 256 |
|  | 37.38 | Morphinan-4,5-epoxy-3,6-di-ol, 6-[7-nitrobenzofurazan-4-yl]amino- | 0.95 | C26H27N5O6 | | 505 |
|  | | | | | | |
| 1 | 7.28 | 9,12,15-octadecatrienoic acid, 2-[(trimethylsilyl)oxy]-1-[[(trimethylsilyl)oxy]methyl]ethyl ester, | 1.92 | C27H52O4Si2 | | 496 |
|  | 8.50 | 4H-Pyran-4-one, 2,3-dihydro-3,5-dihydroxy-6-methyl- | 4.10 | C6H8O4 |  | 144 |
|  | 9.96 | α-D-glucopyranoside, methyl 2,3-bis-O-(trimethylsilyl)-, cyclic butylboronate | 1.31 | C17H37BO6Si2 | | 404 |
|  | 10.11 | 2-trimethylsiloxy-6-hexadecenoic acid, methyl ester | 1.63 | C20H40O3Si | | 356 |
|  | 10.19 | 3,7,11,14,18-pentaoxa-2,19-disilaeicosane, 2,2,19,19-tetramethyl- | 0.49 | C17H40O5Si2 | | 380 |
|  | 10.31 | glucopyranose-1,2-3,5-di-methylboronate-6-TMS | 2.19 | C11H22B2O6Si | | 300 |
|  | 11.51 | octadecanoic acid, 9,10-epoxy-18-(trimethylsiloxy)-, methyl ester, cis- | 1.25 | C22H44O4Si | | 400 |
|  | 12.23 | niacin, TMS derivative | 0.95 | C9H13NO2Si | | 195 |
|  | 12.35 | benzeneacetic acid, TMS derivative | 1.66 | C11H16O2Si | | 208 |
|  | 12.81 | (2,2-dimethyl-5-[2-(2-trimethylsilylethoxymethoxy)propyl][1,3]dioxolan-4-yl)methanol | 3.01 | C15H32O5Si | | 320 |
|  | 13.14 | 6-dimethyl(trimethylsilyl)silyloxytetradecane | 3.16 | C19H44OSi2 | | 344 |
|  | 14.61 | 9,12,15-octadecatrienoic acid, 2-[(trimethylsilyl)oxy]-1-[[(trimethylsilyl)oxy]methyl]ethyl ester, (Z,Z,Z)- | 2.35 | C27H52O4Si2 | | 496 |
|  | 16.17 | dodecanoic acid, 2,3-bis(acetyloxy)propyl ester | 0.72 | C19H34O6 | | 358 |
|  | 17.36 | l-galactopyranose, 6-deoxy-1,2-bis-O-(trimethylsilyl)-, cyclic methylboronate | 4.29 | C11H22B2O6Si | | 300 |
|  | 19.34 | desulphosinigrin | 1.05 | C13H29BO5Si2 | | 332 |
|  | 21.06 | 2,2-dimethyl-5-[2-(2-trimethylsilylethoxymethoxy)-propyl]-[1,3]dioxolane-4-carboxaldehyde | 18.58 | C10H17NO6S | | 279 |
|  | 22.33 | 10-undecynoic acid, TMS derivative | 10.87 | C15H30O5Si | | 318 |
|  | 22.73 | mannose | 0.85 | C14H26O2Si | | 254 |
|  | 22.98 | hexadecanoic acid, 2,3-dihydroxypropyl ester | 1.51 | C6H12O6 |  | 180 |
|  | 23.21 | undecanoic acid, 11-fluoro-, trimethylsilyl ester | 0.54 | C10H17NO6S | | 279 |
|  | 23.52 | [1,1'-bicyclopropyl]-2-octanoic acid, 2'-hexyl-, methyl ester | 1.05 | C19H38O4 | | 330 |
|  | 24.04 | 17-octadecynoic acid | 2.06 | C14H29FO2Si | | 276 |
|  | 25.18 | cyclopentanetridecanoic acid, methyl ester | 1.71 | C21H38O2 | | 322 |
|  | 26.66 | 9-octadecenoic acid (Z)- | 3.64 | C18H32O2 | | 280 |
|  | 26.80 | pentadecanoic acid | 1.90 | C19H36O2 | | 296 |
|  | 27.71 | palmitic acid, TMS derivative | 10.74 | C18H34O2 | | 282 |
|  | 29.00 | oleic acid | 1.81 | C15H30O2 | | 242 |
|  | 29.32 | 13,16-octadecadiynoic acid, methyl ester | 3.45 | C19H40O2Si | | 328 |
|  | 29.82 | 9-hexadecenoic acid | 1.26 | C18H34O2 | | 282 |
|  | 30.02 | 1,3,5-triazine-2,4-diamine, 6-chloro-N-ethyl- | 1.04 | C18H34O2 | | 282 |
|  | 30.58 | cis-13-octadecenoic acid | 0.58 | C19H30O2 | | 290 |
|  | 30.87 | 1-hexadecanol, 2-methyl- | 2.21 | C16H30O2 | | 254 |
|  | 31.35 | phthalic acid, dodecyl oct-3-yl ester | 0.73 | C18H34O2 | | 282 |
|  | 32.83 | 1H-indol-5-ol, 3-(2-aminoethyl)- | 0.67 | C5H8ClN5 | | 173 |
|  | 33.77 | ethanol, 2-[(2-methyl-2-propenyl)oxy]- | 0.92 | C18H34O2 | | 282 |
|  | 36.67 | panaxydol | 2.25 | C17H36O | | 256 |
|  | 37.12 | 5,8,11-eicosatrienoic acid, (Z)-, TMS derivative | 0.60 | C28H46O4 | | 446 |
|  | 37.37 | α-L-galactopyranoside, methyl 6-deoxy-2-O-(trimethylsilyl)-, cyclic methylboronate | 0.94 | C10H12N2O | | 176 |
|  | | | | | | |
| 2 | 8.54 | 9,10-dideutero octadecanoic acid | 2.78 | C6H12O2 | | 116 |
|  | 10.15 | hexadecanoic acid, methyl ester | 0.88 | C27H52O4Si2 | | 496 |
|  | 11.51 | 9,12-octadecadienoyl chloride, (Z,Z)- | 1.40 | C20H40O3Si | | 356 |
|  | 12.81 | 6,9,12-octadecatrienoic acid, methyl ester | 1.49 | C2H7NO3S2 | | 157 |
|  | 13.16 | 6-octadecenoic acid, methyl ester, (Z)- | 1.51 | C15H28B2O6 | | 326 |
|  | 14.60 | 7-methyl-Z-tetradecen-1-ol acetate | 1.95 | C11H22B2O6Si | | 300 |
|  | 17.34 | 1,1-diphenyl-1-(2-dimethylaminoethyl)-2-butanone | 10.99 | C15H30O5Si | | 318 |
|  | 18.99 | 2-myristynoyl pantetheine | 1.06 | C17H24O2 | | 260 |
|  | 19.34 | α-D-glucopyranoside, methyl 2,3-bis-O-(trimethylsilyl)-, cyclic butylboronate | 0.71 | C23H42O2Si | | 378 |
|  | 20.81 | 2-trimethylsiloxy-6-hexadecenoic acid, methyl ester | 12.32 | C10H17NO6S | | 279 |
|  | 21.02 | 3,7,11,14,18-pentaoxa-2,19-disilaeicosane, 2,2,19,19-tetramethyl- | 12.18 | C11H23BO5Si | | 274 |
|  | 21.29 | glucopyranose-1,2-3,5-di-methylboronate-6-TMS | 0.70 | C13H29BO5Si2 | | 332 |
|  | 22.31 | octadecanoic acid, 9,10-epoxy-18-(trimethylsiloxy)-, methyl ester, cis- | 10.07 | C15H30O5Si | | 318 |
|  | 24.04 | niacin, TMS derivative | 0.83 | C26H46O2Si | | 418 |
|  | 25.09 | benzeneacetic acid, TMS derivative | 0.85 | C30H52O3Si | | 488 |
|  | 25.18 | (2,2-dimethyl-5-[2-(2-trimethylsilylethoxymethoxy)propyl][1,3]dioxolan-4-yl)methanol | 1.61 | C5H8ClN5 | | 173 |
|  | 25.48 | 6-dimethyl(trimethylsilyl)silyloxytetradecane | 0.76 | C18H34D2O2 | | 286 |
|  | 26.65 | 9,12,15-octadecatrienoic acid, 2-[(trimethylsilyl)oxy]-1-[[(trimethylsilyl)oxy]methyl]ethyl ester, (Z,Z,Z)- | 3.37 | C18H32O2 | | 280 |
|  | 26.79 | dodecanoic acid, 2,3-bis(acetyloxy)propyl ester | 3.19 | C17H34O2 | | 270 |
|  | 27.45 | l-galactopyranose, 6-deoxy-1,2-bis-O-(trimethylsilyl)-, cyclic methylboronate | 0.78 | C15H30O2 | | 242 |
|  | 27.70 | desulphosinigrin | 11.52 | C18H34O2 | | 282 |
|  | 29.00 | 2,2-dimethyl-5-[2-(2-trimethylsilylethoxymethoxy)-propyl]-[1,3]dioxolane-4-carboxaldehyde | 1.18 | C19H38O4 | | 330 |
|  | 29.31 | 10-undecynoic acid, TMS derivative | 4.52 | C19H40O2Si | | 328 |
|  | 29.83 | mannose | 2.15 | C18H31ClO | | 298 |
|  | 29.90 | hexadecanoic acid, 2,3-dihydroxypropyl ester | 1.12 | C19H32O2 | | 292 |
|  | 30.01 | undecanoic acid, 11-fluoro-, trimethylsilyl ester | 2.39 | C19H36O2 | | 296 |
|  | 30.57 | [1,1'-bicyclopropyl]-2-octanoic acid, 2'-hexyl-, methyl ester | 0.86 | C19H36O2 | | 296 |
|  | 30.85 | 17-octadecynoic acid | 1.87 | C18H34O2 | | 282 |
|  | 32.82 | cyclopentanetridecanoic acid, methyl ester | 0.83 | C17H32O2 | | 268 |
|  | 33.23 | 9-octadecenoic acid (Z)- | 0.82 | C20H25NO | | 295 |
|  | 33.76 | pentadecanoic acid | 1.04 | C10H12N2O | | 176 |
|  | 36.66 | palmitic acid, TMS derivative | 2.27 | C25H44N2O5S | | 484 |

**Sup. Table S2. Effect of *Commiphora myrrha* gum -resins ethanolic extract (g/L) concentrations on the compositions of Phytochemical compounds prepared from *A. fragrantissima* in-vitro plantlets callus stage.**

| *Commiphora myrrha* (g/L) | RT | **Phytochemical compounds** Name | Area % | Molecular Formula | Molecular Weight |
| --- | --- | --- | --- | --- | --- |
| Control | 7.36 | Thymidine | 2.14 | C10H14N2O5 | 242 |
|  | 8.52 | 4h-pyran-4-one, 2,3-dihydro-3,5-dihydroxy-6-methyl- | 7.07 | C6H8O4 | 144 |
|  | 10.16 | α-d-mannopyranoside, methyl, cyclic 2,3:4,6-bis(butylboronate) | 2.88 | C15H28B2O6 | 326 |
|  | 10.31 | D-mannitol, 1-thiohexyl-1-deoxy- | 1.80 | C12H26O5S | 282 |
|  | 10.81 | 7-ethyl-4-decen-6-one | 2.09 | C12H22O | 182 |
|  | 10.92 | 1-dodecanol, 3,7,11-trimethyl- | 4.00 | C15H32O | 228 |
|  | 11.50 | E-10-dodecen-1-ol propionate | 1.41 | C15H28O2 | 240 |
|  | 12.65 | E-8-methyl-7-dodecen-1-ol acetate | 1.89 | C15H28O2 | 240 |
|  | 12.80 | Melezitose | 2.69 | C18H32O16 | 504 |
|  | 14.59 | 4-methyl(trimethylene)silyloxyoctane | 7.11 | C12H26OSi | 214 |
|  | 15.88 | D-fructose, diethyl mercaptal, pentaacetate | 1.12 | C20H32O10S2 | 496 |
|  | 16.62 | Glucopyranose-1,2-3,5-di-methylboronate-6-TMS | 1.41 | C11H22B2O6Si | 300 |
|  | 16.68 | 3-o-hexopyranosylhex-2-ulofuranosyl hexopyranoside | 0.36 | C18H32O16 | 504 |
|  | 17.35 | 2,2-dimethyl-5-[2-(2-trimethylsilylethoxymethoxy)-propyl]-[1,3]dioxolane-4-carboxaldehyde | 13.12 | C15H30O5Si | 318 |
|  | 19.91 | α-d-galactopyranose, 6-O-(trimethylsilyl)-, cyclic 1,2:3,4-bis(methylboronate) | 1.22 | C11H22B2O6Si | 300 |
|  | 20.81 | Dodecanoic acid, 2,3-bis(acetyloxy)propyl ester | 2.72 | C19H34O6 | 358 |
|  | 21.04 | Desulphosinigrin | 2.50 | C10H17NO6S | 279 |
|  | 22.18 | Hexadecanoic acid, 2,3-dihydroxypropyl ester | 2.28 | C19H38O4 | 330 |
|  | 22.30 | 2-aminoethanethiol hydrogen sulfate (ester) | 1.59 | C2H7NO3S2 | 157 |
|  | 22.95 | 1-nitro-α-d-arabinofuranose, tetraacetate | 1.16 | C13H17NO11 | 363 |
|  | 25.47 | 5,8,11-eicosatrienoic acid, (Z)-, TMS derivative | 0.92 | C23H42O2Si | 378 |
|  | 26.08 | 1,25-dihydroxyvitamin D3, TMS derivative | 0.81 | C30H52O3Si | 488 |
|  | 26.78 | Pentadecanoic acid, 14-methyl-, methyl ester | 4.97 | C17H34O2 | 270 |
|  | 27.68 | N-hexadecanoic acid | 11.63 | C16H32O2 | 256 |
|  | 29.30 | Palmitic acid, TMS derivative | 6.33 | C19H40O2Si | 328 |
|  | 29.82 | Oleic acid | 3.41 | C18H34O2 | 282 |
|  | 30.00 | 9-octadecenoic acid (Z)-, methyl ester | 2.57 | C19H36O2 | 296 |
|  | 30.14 | 11-octadecenoic acid, methyl ester | 0.80 | C19H36O2 | 296 |
|  | 30.56 | Cyclopropanepentanoic acid, 2-undecyl-, methyl ester, trans- | 1.36 | C20H38O2 | 310 |
|  | 30.85 | Trans-13-octadecenoic acid | 2.31 | C18H34O2 | 282 |
|  | 31.33 | Tetradecanoic acid | 0.86 | C14H28O2 | 228 |
|  | 32.29 | 9,12-octadecadienoic acid (Z,Z)-, TMS derivative | 0.88 | C21H40O2Si | 352 |
|  | 36.65 | Tert-hexadecanethiol | 2.59 | C16H34S | 258 |
| 0.5 |  | Methyl 6-oxoheptanoate |  |  |  |
|  | 7.34 | Trans-2-dodecenoic acid | 2.79 | C8H14O3 | 158 |
|  | 7.61 | 2,2,19,19-tetramethyl-3,7,11,14,18-pentaoxa-2,19-disilaeicosane | 0.46 | C10H14N2O5 | 242 |
|  | 8.51 | α-d-glucopyranoside, methyl 2,3-bis-O-(trimethylsilyl)-, cyclic methylboronate | 8.82 | C6H8O4 | 144 |
|  | 9.43 | 5-hydroxymethylfurfural | 0.56 | C12H22O2 | 198 |
|  | 10.15 | 1-ethyl-1-cyclohexyloxy-1-silacyclopentane | 1.48 | C15H28B2O6 | 326 |
|  | 10.29 | Maltose | 1.05 | C17H40O5Si2 | 380 |
|  | 10.38 | Octadecanoic acid, 9,10-epoxy-18-(trimethylsiloxy)-, methyl ester, cis- | 0.25 | C14H31BO6Si2 | 362 |
|  | 10.67 | 3-(2-methoxyethyl)-1-nonanol, TMS derivative | 16.15 | C6H6O3 | 126 |
|  | 11.49 | 4-o-hexopyranosylhexopyranose | 0.63 | C18H32O16 | 504 |
|  | 12.64 | 1,3-propanediol, 2-methyl-2-(1-methylpropyl)-, dicarbamate | 3.35 | C12H24OSi | 212 |
|  | 12.85 | l-galactopyranose, 6-deoxy-1,2-bis-O-(trimethylsilyl)-, cyclic methylboronate | 0.84 | C12H22O11 | 342 |
|  | 14.59 | 9,12,15-octadecatrienoic acid, 2-[(trimethylsilyl)oxy]-1-[[(trimethylsilyl)oxy]methyl]ethyl ester, (Z,Z,Z)- | 3.50 | C12H26OSi | 214 |
|  | 15.69 | 7,9-di-tert-butyl-1-oxaspiro(4,5)deca-6,9-diene-2,8-dione | 0.59 | C22H44O4Si | 400 |
|  | 15.89 | Hexadecanoic acid, methyl ester | 0.64 | C15H34O2Si | 274 |
|  | 16.16 | 1-nonadecene | 0.48 | C18H32O16 | 504 |
|  | 16.60 | Methyl-9,9,10,10-D4-octadecanoate | 0.98 | C12H22O11 | 342 |
|  | 16.64 | Cyclopentane, 1-acetyl-1,2-epoxy- | 1.28 | C11H22B2O6Si | 300 |
|  | 17.34 | Ethanimidothioic acid, 2-(dimethylamino)-N-[[(methylamino)carbonyl]oxy]-2-oxo-, methyl ester | 7.46 | C15H30O5Si | 318 |
|  | 18.98 | 1-methyl-1-n-octyloxy-1-silacyclobutane | 0.48 | C10H20N2O4 | 232 |
|  | 19.33 | D-galactol-ido-octonic amide | 0.47 | C15H30O2 | 242 |
|  | 19.90 | Oxiraneundecanoic acid, 3-pentyl-, methyl ester, cis- | 0.87 | C11H22B2O6Si | 300 |
|  | 20.71 | 1-heptatriacotanol | 2.51 | C10H17NO6S | 279 |
|  | 21.02 | 3-hexadecyloxycarbonyl-5-(2-hydroxyethyl)-4-methylimidazolium ion | 3.83 | C13H29BO5Si2 | 332 |
|  | 22.17 | d-glycero-d-ido-heptose | 2.15 | C19H38O4 | 330 |
|  | 22.30 | Methyl 6-O-[1-methylpropyl]-α-d-galactopyranoside | 2.45 | C11H22B2O6Si | 300 |
|  | 22.90 | 10-undecynoic acid, TMS derivative | 0.39 | C27H52O4Si2 | 496 |
|  | 22.97 | 9-tetradecenoic acid, (E)-, TMS derivative | 0.52 | C12H22O11 | 342 |
|  | 25.47 | 9,10-secochola-5,7,10(19)-trien-24-al, 3-hydroxy-, (3α,5Z,7E)- | 0.55 | C17H37BO6Si2 | 404 |
|  | 26.07 | Hexadecanoic acid, 1-(hydroxymethyl)-1,2-ethanediyl ester | 0.64 | C17H24O3 | 276 |
|  | 26.78 | 2-bromotetradecanoic acid | 5.10 | C17H34O2 | 270 |
|  | 27.42 | 6-octadecenoic acid, methyl ester, (Z)- | 0.44 | C18H34O2 | 282 |
|  | 27.68 | 9-octadecenoic acid, (E)-, TMS derivative | 12.24 | C16H32O2 | 256 |
|  | 29.29 | 7-heptadecene, 1-chloro- | 4.80 | C19H40O2Si | 328 |
|  | 29.80 | Thymidine | 1.93 | C19H38 | 266 |
|  | 30.00 | 4h-pyran-4-one, 2,3-dihydro-3,5-dihydroxy-6-methyl- | 2.42 | C19H36O2 | 296 |
|  | 30.13 | α-d-mannopyranoside, methyl, cyclic 2,3:4,6-bis(butylboronate) | 0.47 | C18H34O2 | 282 |
|  | 30.56 | D-mannitol, 1-thiohexyl-1-deoxy- | 1.11 | C19H34D4O2 | 302 |
|  | 30.83 | 7-ethyl-4-decen-6-one | 2.13 | C18H34O2 | 282 |
|  | 31.33 | 1-dodecanol, 3,7,11-trimethyl- | 0.89 | C2H7NO3S2 | 157 |
|  | 32.28 | E-10-dodecen-1-ol propionate | 0.45 | C30H52O3Si | 488 |
|  | 36.65 | E-8-methyl-7-dodecen-1-ol acetate | 1.85 | C17H36O | 256 |
|  |  | Melezitose |  |  |  |
| 1 | 7.33 | 4-methyl(trimethylene)silyloxyoctane | 4.48 | C10H14N2O5 | 242 |
|  | 7.57 | D-fructose, diethyl mercaptal, pentaacetate | 0.92 | C7H10O2 | 126 |
|  | 8.10 | Glucopyranose-1,2-3,5-di-methylboronate-6-TMS | 0.51 | C7H13N3O3S | 219 |
|  | 8.51 | 3-o-hexopyranosylhex-2-ulofuranosyl hexopyranoside | 10.16 | C6H8O4 | 144 |
|  | 9.93 | 2,2-dimethyl-5-[2-(2-trimethylsilylethoxymethoxy)-propyl]-[1,3]dioxolane-4-carboxaldehyde | 0.37 | C12H26OSi | 214 |
|  | 10.16 | α-d-galactopyranose, 6-O-(trimethylsilyl)-, cyclic 1,2:3,4-bis(methylboronate) | 1.1 | C15H28B2O6 | 326 |
|  | 10.30 | Dodecanoic acid, 2,3-bis(acetyloxy)propyl ester | 1.16 | C17H40O5Si2 | 380 |
|  | 10.71 | Desulphosinigrin | 3.75 | C12H22O | 182 |
|  | 10.80 | Hexadecanoic acid, 2,3-dihydroxypropyl ester | 7.97 | C9H14O3 | 170 |
|  | 11.49 | 2-aminoethanethiol hydrogen sulfate (ester) | 1.07 | C18H32O16 | 504 |
|  | 12.18 | 1-nitro-α-d-arabinofuranose, tetraacetate | 0.25 | C12H22O11 | 342 |
|  | 12.64 | 5,8,11-eicosatrienoic acid, (Z)-, TMS derivative | 2.51 | C12H24OSi | 212 |
|  | 12.83 | 1,25-dihydroxyvitamin D3, TMS derivative | 9.95 | C11H22B2O6Si | 300 |
|  | 14.14 | Pentadecanoic acid, 14-methyl-, methyl ester | 0.84 | C18H32O16 | 504 |
|  | 14.23 | N-hexadecanoic acid | 0.89 | C22H44O4Si | 400 |
|  | 14.59 | Palmitic acid, TMS derivative | 3.08 | C12H26OSi | 214 |
|  | 14.79 | Oleic acid | 0.70 | C8H17NO8 | 255 |
|  | 15.89 | 9-octadecenoic acid (Z)-, methyl ester | 0.38 | C27H52O4Si2 | 496 |
|  | 16.54 | 11-octadecenoic acid, methyl ester | 0.34 | C11H22B2O6Si | 300 |
|  | 16.62 | Cyclopropanepentanoic acid, 2-undecyl-, methyl ester, trans- | 0.49 | C12H26OSi | 214 |
|  | 17.35 | Trans-13-octadecenoic acid | 7.96 | C15H30O5Si | 318 |
|  | 18.90 | Tetradecanoic acid | 0.33 | C23H42O2Si | 378 |
|  | 18.98 | 9,12-octadecadienoic acid (Z,Z)-, TMS derivative | 0.68 | C2H7NO3S2 | 157 |
|  | 21.05 | Tert-hexadecanethiol | 12.07 | C10H17NO6S | 279 |
|  | 22.76 | Methyl 6-oxoheptanoate | 0.21 | C19H38O4 | 330 |
|  | 23.03 | Trans-2-dodecenoic acid | 1.35 | C6H12O6 | 180 |
|  | 23.19 | 2,2,19,19-tetramethyl-3,7,11,14,18-pentaoxa-2,19-disilaeicosane | 0.24 | C10H12N2O | 176 |
|  | 23.70 | α-d-glucopyranoside, methyl 2,3-bis-O-(trimethylsilyl)-, cyclic methylboronate | 0.44 | C15H30O2 | 242 |
|  | 24.76 | 5-hydroxymethylfurfural | 0.21 | C26H30O16 | 598 |
|  | 25.70 | 1-ethyl-1-cyclohexyloxy-1-silacyclopentane | 0.21 | C18H34O2 | 282 |
|  | 26.07 | Maltose | 0.35 | C17H24O3 | 276 |
|  | 26.78 | Octadecanoic acid, 9,10-epoxy-18-(trimethylsiloxy)-, methyl ester, cis- | 2.50 | C17H34O2 | 270 |
|  | 27.69 | 3-(2-methoxyethyl)-1-nonanol, TMS derivative | 9.32 | C16H32O2 | 256 |
|  | 28.98 | 4-o-hexopyranosylhexopyranose | 0.32 | C18H34D2O2 | 286 |
|  | 29.04 | 1,3-propanediol, 2-methyl-2-(1-methylpropyl)-, dicarbamate | 0.37 | C15H30O2 | 242 |
|  | 29.10 | l-galactopyranose, 6-deoxy-1,2-bis-O-(trimethylsilyl)-, cyclic methylboronate | 0.20 | C2H7NO3S2 | 157 |
|  | 29.29 | 9,12,15-octadecatrienoic acid, 2-[(trimethylsilyl)oxy]-1-[[(trimethylsilyl)oxy]methyl]ethyl ester, (Z,Z,Z)- | 2.10 | C19H40O2Si | 328 |
|  | 29.57 | 7,9-di-tert-butyl-1-oxaspiro(4,5)deca-6,9-diene-2,8-dione | 0.57 | C19H38O4 | 330 |
|  | 29.79 | Hexadecanoic acid, methyl ester | 1.45 | C18H34O2 | 282 |
|  | 30.00 | 1-nonadecene | 1.38 | C19H36O2 | 296 |
|  | 30.13 | Methyl-9,9,10,10-D4-octadecanoate | 0.51 | C18H34O2 | 282 |
|  | 30.56 | Cyclopentane, 1-acetyl-1,2-epoxy- | 1.03 | C19H36O3 | 312 |
|  | 30.67 | Ethanimidothioic acid, 2-(dimethylamino)-N-[[(methylamino)carbonyl]oxy]-2-oxo-, methyl ester | 0.15 | C37H76O | 536 |
|  | 30.84 | 1-methyl-1-n-octyloxy-1-silacyclobutane | 2.27 | C16H30O2 | 254 |
|  | 31.33 | D-galactol-ido-octonic amide | 0.88 | C18H34O2 | 282 |
|  | 32.26 | Oxiraneundecanoic acid, 3-pentyl-, methyl ester, cis- | 0.41 | C24H46O2 | 366 |
|  | 33.23 | 1-heptatriacotanol | 0.36 | C30H52O3Si | 488 |
|  | 36.64 | 3-hexadecyloxycarbonyl-5-(2-hydroxyethyl)-4-methylimidazolium ion | 1.23 | C19H36O4 | 328 |
|  |  | d-glycero-d-ido-heptose |  |  |  |
| 2 | 7.34 | Methyl 6-O-[1-methylpropyl]-α-d-galactopyranoside | 1.23 | C8H14O3 | 158 |
|  | 7.39 | 10-undecynoic acid, TMS derivative | 1.30 | C18H32O16 | 504 |
|  | 7.59 | 9-tetradecenoic acid, (E)-, TMS derivative | 1.32 | C24H45N2O3 | 409 |
|  | 8.49 | 9,10-secochola-5,7,10(19)-trien-24-al, 3-hydroxy-, (3α,5Z,7E)- | 6.59 | C6H8O4 | 144 |
|  | 10.14 | Hexadecanoic acid, 1-(hydroxymethyl)-1,2-ethanediyl ester | 1.58 | C17H37BO6Si2 | 404 |
|  | 10.25 | 2-bromotetradecanoic acid |  | C15H28B2O6 | 326 |
|  | 11.49 | 6-octadecenoic acid, methyl ester, (Z)- | 0.86 | C27H52O4Si2 | 496 |
|  | 12.63 | 9-octadecenoic acid, (E)-, TMS derivative | 0.94 | C18H32O16 | 504 |
|  | 12.80 | 7-heptadecene, 1-chloro- | 2.05 | C12H22O11 | 342 |
|  | 12.95 | Thymidine | 0.37 | C22H44O4Si | 400 |
|  | 14.58 | 4h-pyran-4-one, 2,3-dihydro-3,5-dihydroxy-6-methyl- | 4.65 | C12H26OSi | 214 |
|  | 16.65 | α-d-mannopyranoside, methyl, cyclic 2,3:4,6-bis(butylboronate) | 4.55 | C2H7NO3S2 | 157 |
|  | 17.33 | D-mannitol, 1-thiohexyl-1-deoxy- | 10.00 | C15H30O5Si | 318 |
|  | 19.86 | 7-ethyl-4-decen-6-one | 0.59 | C12H22O11 | 342 |
|  | 19.90 | 1-dodecanol, 3,7,11-trimethyl- | 0.63 | C11H22B2O6Si | 300 |
|  | 20.00 | E-10-dodecen-1-ol propionate | 0.30 | C11H22B2O6Si | 300 |
|  | 20.69 | E-8-methyl-7-dodecen-1-ol acetate | 5.59 | C7H14O7 | 210 |
|  | 20.83 | Melezitose | 1.71 | C11H22O6 | 250 |
|  | 21.00 | 4-methyl(trimethylene)silyloxyoctane | 7.06 | C11H23BO5Si | 274 |
|  | 21.29 | D-fructose, diethyl mercaptal, pentaacetate | 0.69 | C15H30O2 | 242 |
|  | 22.26 | Glucopyranose-1,2-3,5-di-methylboronate-6-TMS | 3.20 | C14H26O2Si | 254 |
|  | 22.92 | 3-o-hexopyranosylhex-2-ulofuranosyl hexopyranoside | 1.33 | C10H17NO6S | 279 |
|  | 25.47 | 2,2-dimethyl-5-[2-(2-trimethylsilylethoxymethoxy)-propyl]-[1,3]dioxolane-4-carboxaldehyde | 0.67 | C17H34O2Si | 298 |
|  | 26.07 | α-d-galactopyranose, 6-O-(trimethylsilyl)-, cyclic 1,2:3,4-bis(methylboronate) | 0.34 | C24H36O2 | 356 |
|  | 26.77 | Dodecanoic acid, 2,3-bis(acetyloxy)propyl ester | 4.40 | C17H34O2 | 270 |
|  | 27.42 | Desulphosinigrin | 0.50 | C23H42O2Si | 378 |
|  | 27.66 | Hexadecanoic acid, 2,3-dihydroxypropyl ester | 9.25 | C16H32O2 | 256 |
|  | 28.82 | 2-aminoethanethiol hydrogen sulfate (ester) | 0.25 | C35H68O5 | 568 |
|  | 28.95 | 1-nitro-α-d-arabinofuranose, tetraacetate | 1.11 | C14H27BrO2 | 306 |
|  | 29.10 | 5,8,11-eicosatrienoic acid, (Z)-, TMS derivative | 0.26 | C14H28O2 | 228 |
|  | 29.29 | 1,25-dihydroxyvitamin D3, TMS derivative | 6.86 | C19H40O2Si | 328 |
|  | 29.79 | Pentadecanoic acid, 14-methyl-, methyl ester | 2.29 | C18H34O2 | 282 |
|  | 29.99 | N-hexadecanoic acid | 2.47 | C19H36O2 | 296 |
|  | 30.12 | Palmitic acid, TMS derivative | 1.03 | C19H36O2 | 296 |
|  | 30.55 | Oleic acid | 1.10 | C19H38O2 | 298 |
|  | 30.83 | 9-octadecenoic acid (Z)-, methyl ester | 2.70 | C16H30O2 | 254 |
|  | 31.33 | 11-octadecenoic acid, methyl ester | 0.76 | C19H38O4 | 330 |
|  | 32.27 | Cyclopropanepentanoic acid, 2-undecyl-, methyl ester, trans- | 1.03 | C21H42O2Si | 354 |
|  | 33.34 | Trans-13-octadecenoic acid | 0.61 | C18H34O2 | 282 |
|  | 36.64 | Tetradecanoic acid | 1.90 | C17H33Cl | 272 |
